# Be My Assistant or My Partner? How AI Relational Roles and Goal Distance Impact Consumers’ Intentions to Participate in Collective Sustainable Behavior

**DOI:** 10.3390/bs16091517

**Published:** 2026-08-28

**Authors:** Sinan Li, Kai Chen

**Affiliations:** School of Economics and Management, Beijing Forestry University, Beijing 100083, China; lisinan@bjfu.edu.cn

**Keywords:** collective sustainable behavior, assistant-type AI, partner-type AI, large-distance goal, small-distance goal, identity affirmation, positive emotional resonance

## Abstract

Encouraging broad consumer participation is an important pathway to achieving sustainable development. Current research mostly focuses on individual sustainable behavior, with less attention given to collective sustainable behavior under a shared goal. This work is based on the widespread embedding and use of relational AI in different scenarios, employing three studies to verify how AI relational roles and goal distance jointly influence consumers’ participation intention in collective sustainable behavior. The research found that when an assistant-type AI is matched with a large-distance goal, it is more effective in enhancing consumers’ participation intentions, whereas a partner-type AI is more persuasive when matched with a small-distance goal. Furthermore, sustainable identity affirmation and positive emotional resonance serve a serial mediating role in the aforementioned interaction effect. This work reveals the matching mechanism between AI relational roles and goal distance, expanding the research perspective of relational AI in promoting sustainable behavior and providing practical insights for leveraging AI to optimize collective sustainable behavior communication strategies.

## 1. Introduction

Greenhouse gas emissions, resource scarcity, and ecological degradation have become systemic challenges faced by the entire world. The environmental crisis is not only a problem of natural system degradation but is also deeply embedded in human behavior patterns (Evans, 2019). Relying solely on the dissemination of environmental knowledge or individual moral appeals is insufficient to bridge the gap between sustainable awareness and actual behavior (Kollmuss & Agyeman, 2002; Amel et al., 2017). Therefore, how to stimulate public participation in sustainable actions has become an important topic in the fields of consumer behavior, environmental psychology, and sustainable governance research.

Around this topic, extensive research has been conducted. Existing research on sustainable behavior often focuses on individual-level and private-sphere environmental behavior, such as green purchasing, energy conservation, and low-carbon travel (Ren et al., 2024). These studies found that individual sustainable behavior is not only determined by environmental attitudes but is also influenced by habits, subjective norms, and contextual convenience (Gkargkavouzi et al., 2019; Puzzo & Prati, 2025). However, in the face of complex issues such as climate change and ecological degradation that span regions, time, and subjects, relying solely on individual green choices is insufficient to drive systemic transformation. Some studies have pointed out that individual actions are necessary but not sufficient; collective action is the key pathway to achieving sustainable transformation (Nielsen et al., 2021). Nevertheless, current research has insufficiently focused on how consumers can form the willingness to participate in collective sustainable behavior under a shared goal framing (De Gregorio et al., 2025). Based on this, this work concentrates on collective sustainable behavior and defines it as the behavior of consumers who, based on common environmental goals and public interest demands, are willing to participate, collaboratively promote, or support sustainable development with others. It differs from general individual green behavior, placing greater emphasis on the psychological shift from “Me” to “We” (Valizadeh et al., 2025), embedding actions within group collaboration and common goals. Furthermore, collective sustainable behavior has clear public goods attributes, requiring individuals to invest time, effort, or resources to promote collective public benefits (Niemiec et al., 2020; Bodin, 2017). Therefore, collective sustainable behavior is not merely the simple sum of individual actions but a complex behavior involving shared goals, group identity, social norms, and expectations of public benefits. Collective sustainable initiatives may be initiated by public agencies, nonprofit organizations, or firms, but the present research focuses on their consumer-facing communication function rather than on differences in institutional authority. The primary intended stakeholders are sustainability marketers and campaign managers, who use AI-enabled communication to invite consumers to join collective sustainable initiatives. Therefore, the experimental scenarios represent publicly accessible, community-facing communication channels and do not identify the communicator as a governmental authority or compare trust in government, nonprofit, and commercial organizations.

Despite its increasing importance, public participation in collective sustainable behavior still faces obstacles. Environmental issues are often characterized by long-term effects, complexity, and delayed outcomes, making individuals perceive their own actions as insignificant, thereby weakening their willingness to participate (Langlais et al., 2026). K. Kim et al. (2024) found that goal proximity can influence individuals’ willingness to participate in collective action through perceived behavioral impact and collective outcome expectancy, indicating that whether individual actions are perceived as useful and whether they can yield results in conjunction with collective efforts are important psychological judgments that promote action. In the context of collective goals, goal distance is an important variable influencing participation intentions, and individuals’ responses to goals dynamically change with the progress of the goal (S. C. Huang et al., 2017). H. Dai and Zhang (2019) found that collective projects closer to the goal are more likely to stimulate consumers’ prosocial motivation. Jin et al. (2023) further found that long-range collective goals are more suitable for fact-based appeals, while close-range collective goals are more suitable for affect-based appeals. This indicates that goal distance not only affects goal attainability and action value judgment but also alters information processing methods and persuasion pathways.

Furthermore, with the development of artificial intelligence technology, AI is gradually becoming an important entity in sustainable behavior intervention and consumer communication. Existing research often compares the persuasive differences between AI and human information sources, such as the “word-of-machine effect,” which indicates that consumers show preference differences between AI and human recommenders across different task types (Longoni & Cian, 2022). But with the development of interactive AI and generative AI, relational and social aspects have made AI no longer just a static tool but potentially a communication entity with relational roles, emotional responses, and relational attributes, such as assistants, agents, and companions (Patil et al., 2026). Therefore, the persuasive effects of AI should not be understood solely from the perspective of AI or humans but should further examine the relational expectations and communication functions activated by different AI relational roles. Therefore, we believe that with the widespread application of AI, relational AI will participate in individual interactions in different social contexts and play an important persuasive role in the process of promoting collective sustainable behavior. But how different AI roles perform when conveying different goal framings, and whether their effects show significant differences, still need further verification.

Based on this, this work distinguishes AI relational roles as assistants and partners. Drawing on the research by Jin et al. (2023), we also divide goal distance into large and small levels for research purposes. We believe that AI is not a homogeneous source of information; its functional roles can influence individuals’ understanding and acceptance of information (Igarashi et al., 2026), and AI relational roles and goal distance can interactively influence collective sustainable behavior. Assistant-type AI is more akin to advisors and normative guides, embodying goal setting, directional guidance, and long-term planning. The partner-type AI, on the other hand, can reduce psychological distance through emotional closeness, shared experiences, and immediate support, making it more suitable for conveying small-distance goals that emphasize present actions, immediate participation, and shared experiences. Moreover, we believe that the alignment between AI roles and goal distance does not operate solely through rational judgment; identity and emotion are also key factors driving collective sustainable behavior (Schulte et al., 2020). Identity affirmation can explain how individuals transition from being individuals to becoming members of a sustainable community and make them more likely to engage in collective behavior (Valizadeh et al., 2025). Simultaneously, climate change can evoke complex emotions, which, under certain conditions, may become constructive emotions that promote collective behavior (Marczak et al., 2023). Therefore, positive emotional resonance helps transform abstract sustainability goals into concrete willingness to participate. Further, identity affirmation and positive emotional resonance may occur as continuous psychological responses (Luo et al., 2020). Thus, we hypothesize that identity affirmation and positive emotional resonance are important mediating factors and play a serial mediating role.

This work has significant theoretical value and practical insight. In terms of theoretical contributions, we place collective sustainable behavior within the framework of common goals, public goods, and group collaboration, addressing the shortcomings of existing sustainable behavior research that overly focuses on individual green behavior while relatively neglecting collective participation intentions. Moreover, it advances research on AI-mediated persuasion and regulatory fit by demonstrating that the effectiveness of an AI communicator depends on the fit between its relational role and the psychological requirements created by goal distance. Regulatory fit research suggests that when the manner of engagement sustains individuals’ current goal orientation or concerns, people experience a sense of “fit right,” which strengthens engagement and persuasion (Avnet & Higgins, 2006; Cesario et al., 2004). Extending this logic, this research conceptualizes assistant-type and partner-type AI as two relationally embedded modes of goal support and shows that their effectiveness changes dynamically with the distance remaining to a collective goal. Furthermore, this work introduces a chain mediation path of identity affirmation and positive emotional resonance; the serial mediation provides a contextual elaboration of the identity-value model (Berkman et al., 2017), constructing an integrated explanatory framework that influences participation in collective sustainable behavior. In practice, this work provides insights for companies and marketers in designing AI-driven sustainable behavior interventions. By dynamically matching communication strategies based on AI relational roles and goal distance, it can more effectively stimulate consumers’ intentions to participate in collective sustainable behavior, thereby promoting ecological environmental protection and the achievement of sustainable development goals.

## 2. Literature Review and Hypothesis Development

### 2.1. Collective Sustainable Behavior

Collective sustainable behavior refers to individuals participating in actions with sustainable goals as members of a group, community, or social action participants in order to address public issues such as climate change, ecological degradation, and pollution management (Hertel et al., 2026). Examples include participating in community environmental protection projects, supporting collective green initiatives, or assisting in achieving collective carbon reduction goals. Unlike traditional sustainable behavior, which emphasizes individual-level green purchasing, energy conservation, and low-carbon living, collective sustainable behavior places greater emphasis on the transformation of actions from “Me” to “We.” This means that individuals not only focus on whether their own actions are environmentally friendly but also on whether their actions can jointly create a broader social and ecological impact with others (Valizadeh et al., 2025). Issues such as climate change, resource scarcity, and ecological degradation are characterized by their long-term, complex, and public nature. Individual actions alone are insufficient for bringing about systemic changes; therefore, cooperation and social mobilization are needed to create more substantial sustainable impacts (Ardoin & Bowers, 2025). At the same time, climate change behavior is not merely the result of environmental attitudes but is also influenced by social motivations such as belonging, understanding, self-improvement, and trust (Brick et al., 2021). This further illustrates that collective sustainable behavior is a form of behavior embedded in social relationships and shared goals.

Existing research has demonstrated the importance of collective sustainable behavior from the perspectives of social psychology, community governance, green consumption, and sustainable transformation. The research indicates that collective behavior is not an irrational group response, but rather a social action process shaped by shared identity, group norms, and collective narratives (Drury, 2020). Collective narratives, as meta-stories shared by the group, can provide members with value frameworks and behavioral norms, thereby influencing cooperation, prosocial actions, and participation in social movements (Bliuc & Chidley, 2022). In the context of sustainability, research on sustainable communities, sustainable tourism, and sustainable environmental governance reveals that factors such as social identity, collective efficacy, participative efficacy, trust, social networks, and subjective norms all influence individuals’ willingness to participate in collective behavior (Jamšek & Culiberg, 2025). This indicates that collective sustainable behavior not only depends on individual environmental attitudes but also relies on group relationships, shared identity, and collaborative mechanisms (Plechatá et al., 2025a). However, consumers should not be treated as psychologically homogeneous. Prior environmental knowledge and involvement can shape baseline motivation, information elaboration, and responses to sustainability communications. Recent evidence indicates that environmental knowledge does not necessarily exert a simple direct effect on sustainable behavior; instead, it may operate through consumer attitudes, perceived environmental responsibility, and private-sphere pro-environmental behavior, while its effects may also depend on environmental awareness (Rousta & Allaf Jafari, 2024; Trinh et al., 2025). Sustainability knowledge and involvement have also been shown to condition how consumers translate perceived environmental benefits and green information frames into behavioral intentions (Prados-Peña et al., 2024; Zou et al., 2025). Consumers with greater knowledge or involvement may possess established interpretive frameworks and attend more closely to the diagnostic content of environmental messages, whereas less knowledgeable or less involved consumers may depend more strongly on explanatory guidance, efficacy information, or relational encouragement. In addition, past pro-environmental behavior can carry over to subsequent environmental actions through environmental self-identity and subjective norms, although such spillover is context-dependent (Teng et al., 2025). Green skepticism can likewise alter how consumers evaluate the credibility and perceived value of environmental claims and has been linked to trust, product evaluation, and responses to green communication (Higueras-Castillo et al., 2024; Sivapalan et al., 2024).

At the same time, collective sustainable behavior can also be understood from perspectives of practice and intervention. Practice theory posits that sustainable transformation not only relies on formal organizations or social movements but also manifests as dispersed, continuous collective activities in daily life, where people gradually drive social change through shared arrangements and social interactions (Welch & Yates, 2018). Research shows that collective public commitment can help people develop a more conscious environmental awareness, promote behavioral changes, and sustain certain sustainable lifestyles over the long term (Lindemann-Matthies et al., 2021). Moreover, through choice architecture designs such as default options, eco-labels, and sustainable prompts, it is possible to promote individual well-being and collective sustainability without restricting individual freedom (Dorigoni & Bonini, 2025), thereby effectively utilizing sustainable nudging. These studies reveal that collective sustainable behavior can be stimulated and continuously reinforced through commitments, social support, behavioral prompts, and contextual stimuli (Sheng et al., 2022).

Some research has laid a solid foundation for understanding collective sustainable behavior, but studies have mostly focused on social movements, public policy, educational interventions, and agricultural environmental projects (Ardoin & Bowers, 2025; Sander et al., 2024), with less attention given to collective sustainable behavior in the contexts of digital marketing and AI interactions. Moreover, although existing research has noted goal progress, public commitment, and prosocial behavior (Fishbach et al., 2011; Koo & Fishbach, 2012), there is still a lack of systematic explanations of how different AI relational roles and goal distance jointly influence willingness to engage in collective sustainable behavior. Therefore, this work explores the impact of AI relational roles and goal distance on the willingness to participate in collective sustainable behavior, further revealing the psychological mechanisms behind the behavior. It expands the application boundaries of collective sustainable behavior in AI marketing contexts and provides a new theoretical perspective for understanding the shift in consumers from individual green actions to collective sustainable participation.

### 2.2. The Interaction Effect of AI Relational Roles and Goal Distance

As the application of interactive AI in consumer and social behavior intervention scenarios continues to expand (Plechatá et al., 2025b), AI is no longer just a technical tool that passively executes tasks but is gradually being understood by consumers as an agent capable of assisting with goal setting, emotional support, and continuous interaction (Pantano & Scarpi, 2022). A number of practice-based theoretical studies are also continuing to broaden our understanding of AI. The Computers Are Social Actors (CASA) theory posits that individuals respond to computers in social ways (Nass et al., 1994; Xu et al., 2022), while the AI-mediated communication (AIMC) theory examines how AI intervenes in and alters information transfer, social relationships, and communication outcomes (Hancock et al., 2020; Hennighausen et al., 2025). These theories provide important theoretical underpinnings for further explaining human–computer interaction. In this trend, relational AI has gradually become an important communication role in human–computer interaction design and practice. In this research, AI relational roles refer to the socially interpretable roles through which consumers understand and interact with AI agents in a communication context. Rather than treating AI merely as an information source, consumers may respond to AI as a relationship-like social actor with distinct role expectations and communication functions (Fournier, 1998; Aggarwal, 2004; M. H. Huang & Rust, 2021). By endowing AI with a human-like appearance, language, or social characteristics, consumers find it easier to perceive AI as an interactive entity (Moriuchi, 2021; Husain & Prentice, 2025; Park et al., 2026). This helps to enhance social presence and psychological interaction, thereby increasing consumers’ acceptance of AI-recommended content (Wang et al., 2025). Research on human–AI partnerships indicates that different AI relational roles have distinct functional roles in goal pursuit (Park & Ahn, 2024). For instance, AI assistants or AI counselors emphasize information processing, efficiency enhancement, and task execution, while partner-type AI focuses more on emotional and psychological well-being goals, forming close social-distance connections through continuous interaction (Patil et al., 2026). Within this framework, we classify AI relational roles into two distinct types: assistant and partner. This distinction is consistent with research showing that consumers evaluate humanlike nonhuman agents according to role schemas, relationship norms, and social cues (Aggarwal & McGill, 2007; Mende et al., 2019). Assistant and partner can be understood as two types of AI relational roles with differentiated psychological functions. Assistant-type AI is more akin to supporters with guiding, analytical, and normative characteristics, especially in non-programmatic situations that require value judgments and behavioral guidance. In such contexts, AI’s cognitive abilities can reduce algorithm aversion by enhancing mind perception, making users more willing to see them as collaborators rather than mere tools (L. Zhang et al., 2025). In contrast, partner-type AI is defined as interactive partners capable of providing empathy and emotional support, establishing and maintaining close connections. They emphasize companionship and relationship maintenance, which helps improve emotional experiences, such as reducing feelings of loneliness (De Freitas et al., 2025). The differences in practical guidance and emotional empathy among different AI relational roles will significantly affect their support effectiveness (Ricon, 2025). Therefore, assistant-type AI and partner-type AI represent two distinct relational roles: goal guidance and emotional support, respectively (Prentice & Nguyen, 2021; El Hedhli et al., 2023).

Moreover, in collective behavior, goal distance also affects individuals’ judgments of action value and feasibility. Goal distance refers to consumers’ perceived distance between the current state of a collective goal and its desired end state (Kivetz et al., 2006). In collective sustainable behavior, a large-distance goal indicates that the collective target is still far from completion and therefore highlights long-term value, future consequences, and the need for continued contribution. A small-distance goal indicates that the collective target is close to completion and therefore highlights immediacy, attainability, and the visible impact of current participation (Jin et al., 2023). Research on goal pursuit indicates that individuals typically focus more on “Can I achieve the goal?” in the early stages of goal pursuit, but as the goal approaches completion, they shift their attention to “When will I achieve the goal?” and whether their actions can effectively close the remaining gap (S. C. Huang et al., 2017). Koo and Fishbach (2008) pointed out that when individuals focus on goal commitment, the completed progress can reinforce the belief that, in the early stages of a goal, emphasizing the small area that has been completed can better stimulate goal-seekers’ actions; in the later stages, emphasizing the small area that remains can better drive completion (Koo & Fishbach, 2012). In the context of collective goals, the role of goal distance is more complex because individuals do not control goal progress alone; instead, they need to consider others’ contributions, group progress, and their own influence to decide whether to participate. Y. Kim and Reeck (2019) found in environmental waste reduction behaviors that the presentation of collective goal progress significantly affects participation intentions. In the early stages of the goal, emphasizing that a large portion still needs to be completed can increase participation; in the later stages, emphasizing that a large portion is already completed can enhance participation intentions. Due to social norms and goal value signaling of others’ contributions, collective goals are more likely to follow the large-area logic, unlike the small-area logic in individual goals. Specifically, in the early stages of the goal, emphasizing the large contributions that still need to be completed, and in the later stages, emphasizing the large contributions that have already been completed, can more effectively enhance the willingness to participate (Y. Kim & Reeck, 2019). This indicates that collective sustainable behavior is not only driven by the importance of environmental issues but is also influenced by goal distance, goal progress, and cues from others and individual contributions (Jin et al., 2023; Keller et al., 2022).

Although AI relational roles and goal distance represent two conceptually distinct factors, they converge in determining how consumers interpret and respond to an AI-delivered collective goal. AI relational roles are not merely labels attached to a communicator; rather, they signal different modes of support and activate corresponding expectations regarding how the AI should facilitate goal pursuit (M. H. Huang & Rust, 2021). Consumers evaluate such AI roles more favorably when their expressed characteristics are congruent with the human role schema activated by the interaction context (Aggarwal & McGill, 2007). Goal distance, in turn, changes the psychological demands of persuasion. When a collective goal remains far from completion, consumers face greater uncertainty and require diagnostic information to assess the value and feasibility of participation; when the goal is close to completion, consumers become more responsive to affective engagement and the immediate possibility of contributing to goal attainment (Jin et al., 2023). Therefore, the persuasive effect of an AI relational role should depend on whether its characteristic mode of support addresses the psychological demands created by the current goal distance. In this work, we consider that there is an interaction effect between AI relational roles and goal distance, and their alignment can promote consumers’ collective sustainable behavior participation intentions (Jamšek & Culiberg, 2025).

Regulatory fit theory provides a theoretical explanation for the interaction between AI relational roles and goal distance. The theory suggests that persuasion becomes more effective when the means of goal pursuit fits the psychological orientation activated by the goal because such fit makes the message feel right and increases its perceived value (Avnet & Higgins, 2006). In this research, goal distance activates different psychological requirements. According to construal level theory, large-distance goals are more abstract, future-oriented, and uncertainty-laden, requiring cognitive elaboration, goal interpretation, and planning support (Trope & Liberman, 2010; Humphreys et al., 2021). Assistant-type AI fits these requirements because it emphasizes guidance, analysis, and goal management. In contrast, small-distance goals are closer, more immediate, and action-ready, requiring emotional engagement, psychological closeness, and shared participation (Carter et al., 2020). Partner-type AI fits these requirements because it emphasizes companionship and acting together. Therefore, the interaction between AI relational roles and goal distance can be understood as a regulatory fit effect: assistant-type AI creates a better fit with large-distance goals, whereas partner-type AI creates a better fit with small-distance goals.

Based on the above logic, assistant-type AI could have higher compatibility with large-distance goals. In collective sustainable behavior, large-distance goals usually refer to long-term, abstract, and macro sustainable visions, such as ecological improvement or future societal benefits. Although these goals have high value, due to long time frames and the difficulty in perceiving individual impact, consumers may face cognitive doubts about the significance of their personal actions. Assistant-type AI is precisely capable of addressing this issue; its guidance, professionalism, and planning functions can help consumers understand the value of long-term goals, strengthen goal commitment, and embed individual participation within a broader framework of collective action. Even in the case of highly complex goals, AI can also enhance the perception of action effectiveness through its expertise and planning abilities (Patil et al., 2026). Therefore, when collective sustainable behavior is framed as a large-distance goal, assistant-type AI enhances the willingness to participate by explaining why this large-distance goal is worth pursuing and how individual actions serve the collective future.

In contrast, small-distance goals typically have immediacy, specificity, and visibility of action. These goals are more likely to stimulate immediate action, but the key lies in lowering the participation threshold, enhancing instantaneous emotional investment, and reinforcing the experience of acting together with others. The companionship, empathy, and emotional characteristics of partner-type AI can precisely activate this proximal engagement motivation. Partner-type AI, by making individuals feel listened to, supported, and understood, can provide psychological resources similar to social interactions (De Freitas et al., 2025). These companion-oriented roles are more likely to evoke emotional closeness and responses (Manoli et al., 2026). In the context of small-distance goals, consumers are faced with a specific, immediate, and actionable invitation. At this point, it is necessary to reduce action resistance through warmth and companionship. This companionship and closeness help consumers understand collective sustainable behavior as an action goal that can be participated in, felt, and accomplished together in the present moment. Therefore, the combination of partner-type AI and small-distance goals is more likely to enhance willingness to participate through emotional support and immediate resonance.

Based on the above analysis, it can be inferred that AI relational roles and goal distance do not independently affect the willingness to participate in collective sustainable behavior, but rather there is a matching effect between role features and goal structure. Thus, we propose the following hypothesis:

**H1.**  *There is an interaction effect between AI relational roles and goal distance, as follows:*

**H1a.**  *When assistant-type AI highlights a large-distance goal (vs. a small-distance goal), consumers show higher participation intention in collective sustainable behavior.*

**H1b.**  *When partner-type AI highlights a small-distance goal (vs. a large-distance goal), consumers show higher participation intention in collective sustainable behavior.*

### 2.3. The Mediating Role of Identity Affirmation

Identity affirmation refers to the psychological process in which an individual perceives a certain behavior, goal, or external information as consistent with their self-concept, value beliefs, and ideal self in a specific context, thereby confirming how they should act (Confente et al., 2020). In this work, sustainable identity affirmation refers to the psychological process where consumers, when faced with collective sustainable behavior initiatives, perceive themselves as individuals who care about the environment, support sustainable development, and are willing to contribute, thereby interpreting their participation as actions that align with their self-definition and value commitments (Balundė & Perlaviciute, 2023; Li et al., 2025). Sustainable identity reflects the extent to which individuals integrate green and environmental values into their self-concept and can significantly influence their willingness to engage in green consumption and pro-environmental behaviors (Becerra et al., 2023; Fang et al., 2026). The identity-value model also reveals that when a behavior is perceived as identity-relevant, it will gain higher subjective value, making it easier to choose and execute (Berkman et al., 2017). When external information aligns with the consumer’s sustainable self-identity, they confirm an identity consistent with their own values. Alam et al. (2025) pointed out that consumers with a strong environmental self-identity affirmation are more likely to view the purchase of remanufactured products as a way to express and reinforce their environmental identity. Therefore, in this work, the matching of AI relational roles with goal distance can also influence the willingness to participate in collective sustainable behavior by activating sustainable identity affirmation.

Specifically, the combination of assistant-type AI and large-distance goals is more likely to activate consumers’ sustainable identity through meaning interpretation and goal guidance. Although large-distance goals have a strong sense of value, they may also reduce individuals’ perception of the significance of their own actions due to the psychological distance. Assistant-type AI possesses guiding and analytical characteristics, which can help consumers understand the value of long-term sustainable goals, making them aware that participating in this action reflects their commitment to sustainable development, further making it easier to enhance consumers’ sustainable identity affirmation, thereby increasing their willingness to participate in collective sustainable behavior. In comparison, the combination of partner-type AI and small-distance goals is more likely to activate consumers’ sustainable identity through immediate interaction and emotional support. Small-distance goals have specificity, immediacy, and action visibility, allowing consumers to directly feel that they are contributing to the collective goal immediately. The companionship, empathy, and emotional characteristics of partner-type AI can enhance the psychological connection between consumers and the action context, allowing them to confirm their membership in collective sustainable behavior during immediate participation. This identity label can strengthen consumers’ identification strength and promote identity-consistent preferences (Shen et al., 2025). Through the affirmation of social identity, consumers are more willing to engage in sustainable behavior (S. Jiang et al., 2022). Based on this, we propose the following hypothesis:

**H2.**  *Identity affirmation mediates the interaction effect of AI relational roles and goal distance.*

### 2.4. The Mediating Role of Positive Emotional Resonance

Positive emotional resonance refers to a positively valenced affective state elicited when an individual encounters information, an object, or a situation that is consistent with personal concerns or values (Wahlen & Stroude, 2023; Yu et al., 2024). In the present research, it refers specifically to the extent to which exposure to a collective sustainability message makes consumers feel emotionally stirred, refreshed, comfortable and relaxed, satisfied and at ease, and happy. Therefore, the construct captures a general positive affective reaction to the message rather than discrete moral or social emotions such as pride, guilt, duty, responsibility, or a sense of mission. Positive affective reactions may nevertheless facilitate engagement and behavioral intentions by making an advocated action feel emotionally rewarding, pleasant, and personally meaningful (Jang & Namkung, 2009; Sharma et al., 2023).

Consumer emotion research indicates that positive emotional resonance plays a key role in consumer engagement and the formation of behavioral intentions (Wei & Prentice, 2022; Sharma et al., 2023). In the field of sustainable behavior, emotions are an important mechanism driving action (Kovács et al., 2024; Sadiq et al., 2026). Emotions can significantly influence the intention to avoid pollution and to purchase green products (Liang et al., 2019). These emotions are not only internal self-evaluation responses but also have social evaluation functions, indicating that sustainable behavior is not solely driven by rational cognition but is also influenced by emotions. This perspective provides a deeper emotional mechanism explanation for this research. We believe that participating in collective sustainable behavior brings general positive emotions. This emotional transformation and internalization by consumers can further promote their behavioral intentions (Lima et al., 2024).

Specifically, when consumers face large-distance goals, they are more likely to perceive that individual actions can contribute to future environmental and collective well-being. Their sense of emotional appeals toward environmental issues and future concerns is more likely to be awakened; it will be easier to stimulate their intention to act (Ganassali & Ganassali, 2025). When consumers face small-distance goals, these goals emphasize actionable and perceivable features, which can lower the participation threshold, thereby making them more likely to trigger immediate and positive emotional resonance. This positive emotion can significantly enhance the persuasive effect and increase the willingness to participate in behavior (Kao, 2019). Therefore, in the context of small-distance goals, conveying information in a credible and warm manner is more likely to evoke immediate positive emotional resonance (K. Jiang et al., 2024; J. Dai & Gong, 2024), thereby increasing consumers’ willingness to engage in collective sustainable behavior. Based on the above deductions, we propose the following hypothesis:

**H3.**  *Positive emotional resonance mediates the interaction effect of AI relational roles and goal distance.*

### 2.5. The Serial Mediating Role of Identity Affirmation and Positive Emotional Resonance

In addition to their respective mediating roles, identity affirmation and positive emotional resonance may jointly constitute a serial mediation pathway. Identity affirmation reflects a cognitive appraisal of whether participation in a collective sustainable initiative is consistent with an individual’s self-concept and value commitments. When the communication makes a sustainable self-definition more salient, participation may acquire greater subjective value and personal meaning because the proposed action is perceived as affirming who the consumer is or aspires to be (Berkman et al., 2017; Confente et al., 2020). This identity-consistent appraisal may be associated with a more positive emotional response to the initiative because an action that affirms valued aspects of the self may be experienced as more encouraging, emotionally rewarding, and personally meaningful. Positive emotional resonance may subsequently be associated with stronger participation intention by increasing the affective appeal of the proposed collective action. This ordering provides the rationale for examining identity affirmation and positive emotional resonance as serial mediators. However, it does not imply that all consumers necessarily experience these responses as a fixed temporal sequence. Rather, the proposed ordering represents a theoretically informed explanatory model in which identity-related appraisal is expected to be associated with subsequent positive affective evaluation. The empirical analysis examines whether the observed pattern of associations is consistent with this proposed pathway; it does not establish its temporal or causal ordering.

The identity-value model provides the theoretical foundation for this chain mechanism. This model posits that when a behavior aligns with an individual’s core identity, this identity association enhances the subjective value of that behavior, thereby making the individual more likely to choose that behavior (Berkman et al., 2017; Helinski et al., 2025). When the match between AI roles and the goal distance provides external stimulation, identity affirmation and positive emotional resonance occur as continuous psychological responses (Luo et al., 2020). When identity affirmation is activated, the identity label can enhance the subjective value of goal-related behaviors. If a behavior is perceived by the individual as related to their self-identity, it is more likely to dominate in the integration process and be chosen (O’Leary et al., 2017). Based on self-identity theory, Alam et al. (2025) also point out that individuals tend to adopt behaviors consistent with their self-concept. Therefore, when consumers perceive that participating in collective sustainable behavior reflects their identity as a sustainable actor, the behavior no longer remains just an external initiative but becomes a part of self-expression and value realization. Research shows that a sustainable self-identity can enhance behavioral intentions and perceived value (Becerra et al., 2023), and emotional perception can further drive the intention for sustainable behavior (Liang et al., 2019; Yu et al., 2024). This enhancement of identity value further provides a foundation for positive emotional resonance.

In this work, this chain mechanism aligns with the “cognition–emotion–behavior” pathway. This pathway emphasizes that cognitive images can influence emotional images, and both further affect behavioral intentions (Yoon & Kim, 2025). This process can be reflected in two types of matches. On the one hand, in the combination of assistant-type AI and a large-distance goal, through goal interpretation and long-term meaning construction, assistant-type AI helps consumers affirm that they are sustainable actors who care about the future ecology and support collective well-being. This identity affirmation further enhances consumers’ emotional investment in long-term goals, thereby increasing their willingness to participate. On the other hand, under the combination of partner-type AI and small-distance goals, the partner-type AI, through companionship and instant empathy, helps consumers confirm that they are becoming part of a collective sustainable action. This immediate identity affirmation further stimulates positive emotional resonance, making consumers feel supported and encouraged and experience the sense of action of participating together with others, thereby increasing their participation intentions. Based on the above reasoning, we propose the following hypothesis:

**H4.**  *Identity affirmation and positive emotional resonance play a serial mediating role in the interaction effect of AI relational roles and goal distance.*

**Figure 1:**
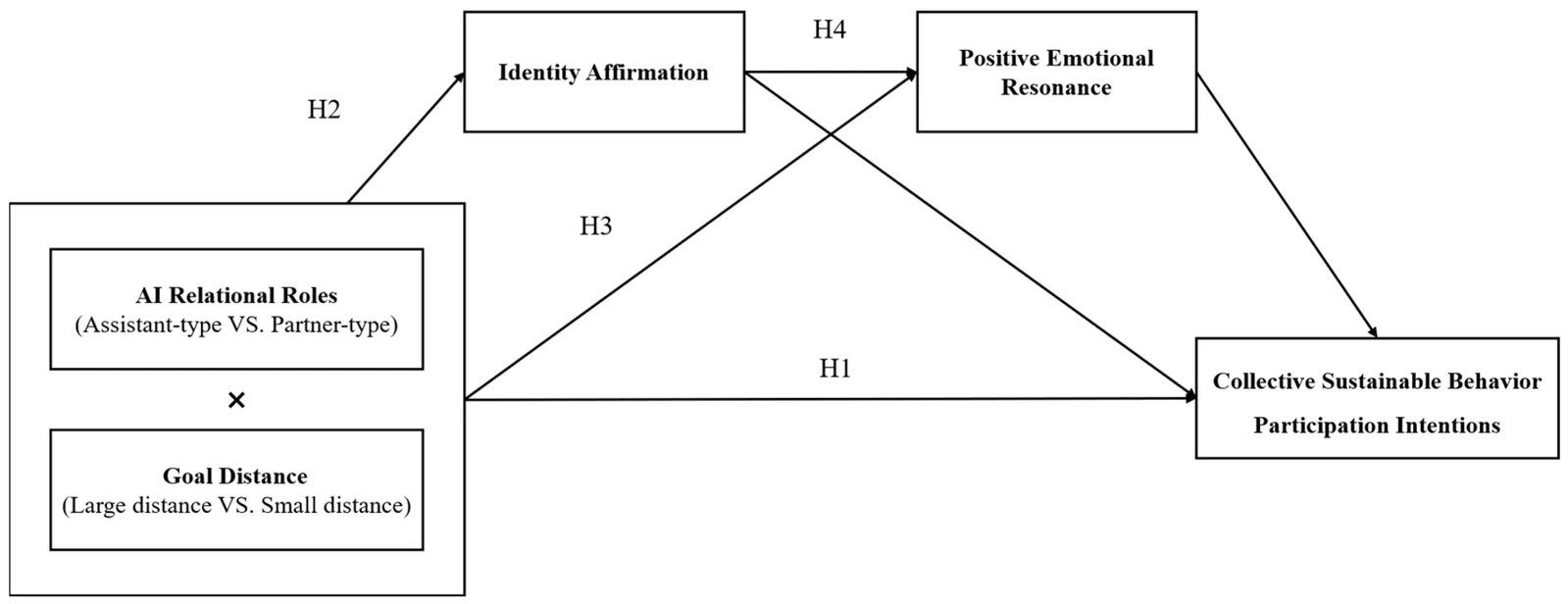
Research model.

We have drawn a conceptual model to visually present research hypotheses and relationships between variables (Figure 1).

## 3. Study 1: Community Resource-Circulation Center Initiative

### 3.1. Experimental Design

Study 1 aims to investigate the interaction between AI relational roles and goal distance, specifically testing Hypothesis 1. We adopted a 2 (AI relational roles: assistant-type vs. partner-type) × 2 (goal distance: large vs. small) between-subjects factorial design.

In Study 1, we designed a community resource-circulation center initiative to compare consumers’ participation intentions with different stimuli by setting AI roles and goal distances. For the design of AI relational roles, we presented them using a combination of images and text, employing the same image and distinguishing different roles through text introductions to avoid interference from varying external image preferences (see Appendix A). The identical image was deliberately retained across the assistant-type and partner-type AI conditions so that facial features, attire, color composition, human likeness, and pre-existing avatar familiarity could not vary with the textual role manipulation. This control is important because visual appearance and conversational style can jointly shape social presence, trust, satisfaction, and visual attention (Park & Ahn, 2024), while avatar realism and familiarity can also influence trust and behavioral responses (J. Chen et al., 2024; Song & Shin, 2024). The design therefore prioritizes internal validity by attributing between-condition differences to the role description rather than to a simultaneous change in visual identity. At the same time, the textual manipulation should be understood as a composite presentation of AI relational roles. The assistant-type and partner-type conditions differed in their role labels, self-descriptions, and communication styles to align with the styles and characteristics of corresponding roles in real life, thereby enhancing the realism of the experimental materials. Moreover, the manipulation check was set to assess whether participants recognized the intended assistant or partner role and did not separately assess perceived warmth, competence, and relational closeness. The design for goal distance draws on the research of Jin et al. (2023), using two different frameworks that emphasize the expressive characteristics of “large-distance” and “small-distance.” We set a goal for the execution and implementation of collective behavior, namely, “the plan will be implemented once 1000 participants join.” By describing the current number of participants, we indicate the distance to the established goal, thereby stimulating participants’ perception and judgment of the goal’s proximity. We described the “large-distance goal” framing as follows: “We proposed to build a community resource-circulation center, aiming to call on 1000 people to participate. Currently, 187 people have joined. As long as we reach 1000 people, the community will build a center for the exchange and circulation of unused clothes, books, and toys.” We described the “small-distance goal” framing as follows: “We proposed to build a community resource-circulation center, aiming to call on 1000 people to participate. Currently, 813 people have joined. As long as we reach 1000 people, the community will build a center for the exchange and circulation of unused clothes, books, and toys.”

In order to test whether the experimental manipulation was successful, we specifically designed test items for this purpose. After viewing the stimulus material and answering questions related to participating in the resource-circulation center initiative, participants needed to respond to the 7-point scale for manipulation checks (1 = strongly disapprove, 7 = strongly approve). The items related to the AI relational roles include (1) “I received the information from an AI assistant”; (2) “The above information comes from the assistant-type AI’s recommendation profile”; (3) “I received the information from an AI partner”; and (4) “The above information comes from the partner-type AI’s recommendation profile.” The measurement items for goal distance include (1) “This resource-circulation center initiative currently has a small number of participants”; (2) “This resource-circulation center initiative is currently far from reaching its goal”; (3) “This resource-circulation center initiative currently has a large number of participants”; and (4) “This resource-circulation center initiative is currently close to reaching its goal.” We tested the differences in participants’ judgments and evaluations after being exposed to different materials in various experimental contexts to verify the effectiveness of the manipulation.

Participants in Study 1 were recruited through Credamo, a professional online research platform. After providing informed consent, participants were randomly assigned by the survey system with equal probability to one of the four experimental conditions. Questionnaire forwarding and multiple submissions were prohibited. A total of 260 responses were received. We sequentially excluded nine responses because of patterned responses (such as ‘Z’-shaped options); the final analytic sample comprised 251 participants (N_female_ = 62.95%, M_age_ = 29.64, SD = 9.09).

### 3.2. Experimental Procedure

First, we designed a hypothetical scenario and guided the participants to imagine themselves in that scenario. We provided the following text: “Please imagine the following scenario: The online platform in your community has embedded an AI chatbot to improve service quality, which can recommend more targeted information to you and engage in daily conversations with you. Recently, the platform has been promoting knowledge related to sustainable behavior, and you saw the following information.” They were then randomly assigned to 1/4 experimental conditions.

In this experiment, we provided background and significance about the resource-circulation center initiative to enhance participants’ sense of actual involvement. For example, we mentioned, “Many households have a lot of idle clothes, books, and toys. These items still have value but are often left unused for long periods or even directly discarded, leading to resource waste. In order to promote resource recycling and build a more sustainable community, we need to work together.” After reading the stimulus information corresponding to their group, participants needed to respond to the 7-point scale regarding their participation intention in the resource-circulation center initiative (1 = strongly disapprove, 7 = strongly approve) with one of the following options: (1) “It is very likely that I will participate in the above collective initiative”; (2) “I believe the above collective initiative is worth participating in”; (3) “I would recommend and encourage my friends to participate in the above initiative”; (4) “I want to participate in the above initiative”; (5) “I will try to participate in the above initiative”; and (6) “I plan to participate in the above initiative immediately” (adapted from Li et al., 2026 and X. Zhang et al., 2024). Subsequently, participants answered the manipulation check items, including judgments on the AI relational roles and goal distance. Finally, they answered questions related to demographic variables.

### 3.3. Results

We first examined whether the experimental manipulation was successful. The results of the paired-sample *t*-test showed that when participants receive information from an assistant-type AI, they are more likely to recognize AI as an assistant rather than a partner (M_assistant_ = 5.86, SD = 1.00; M_partner_ = 3.55, SD = 1.87, *p* < 0.001); conversely, when they receive information from a partner-type AI, they are more likely to recognize the AI as a partner (M_assistant_ = 3.82, SD = 2.05; M_partner_ = 5.82, SD = 1.15, *p* < 0.001). Regarding the judgment of goal distance, when participants received information emphasizing large-distance goals, their ratings for the large dimension were higher (M_large_ = 5.80, SD = 1.10; M_small_ = 2.38, SD = 1.29, *p* < 0.001); conversely, when they received information emphasizing small-distance goals, their ratings for the small dimension were higher (M_large_ = 2.61, SD = 1.41; M_small_ = 5.60, SD = 1.27, *p* < 0.001). The above test results indicated that Study 1 successfully manipulated the AI relational roles and goal distance.

Because both experimental factors were manipulated between subjects, manipulation effectiveness was supplemented using independent-samples Welch’s *t*-tests comparing the relevant ratings across the corresponding assigned conditions. Participants assigned to the assistant-type AI condition rated the communicator as more assistant-like (M = 5.86, SD = 1.00) than participants assigned to the partner-type AI condition (M = 3.82, SD = 2.05; Welch’s t(181.55) = 10.03, *p* < 0.001, Cohen’s d = 1.26). Participants assigned to the partner-type AI condition rated the communicator as more partner-like (M = 5.82, SD = 1.15) than participants assigned to the assistant-type AI condition (M = 3.56, SD = 1.87; Welch’s t(206.02) = 11.53, *p* < 0.001, Cohen’s d = 1.46). Regarding goal distance, participants assigned to the large-distance condition reported stronger large-distance perceptions (M = 5.80, SD = 1.10) than those assigned to the small-distance condition (M = 2.61, SD = 1.41; Welch’s t(233.56) = 19.97, *p* < 0.001, Cohen’s d = 2.52). Conversely, participants assigned to the small-distance condition reported stronger small-distance perceptions (M = 5.60, SD = 1.27) than those assigned to the large-distance condition (M = 2.38, SD = 1.29; Welch’s t(248.96) = 19.95, *p* < 0.001, Cohen’s d = 2.52). These results confirmed that the manipulations of AI relational roles and goal distance were successful.

**Figure 2:**
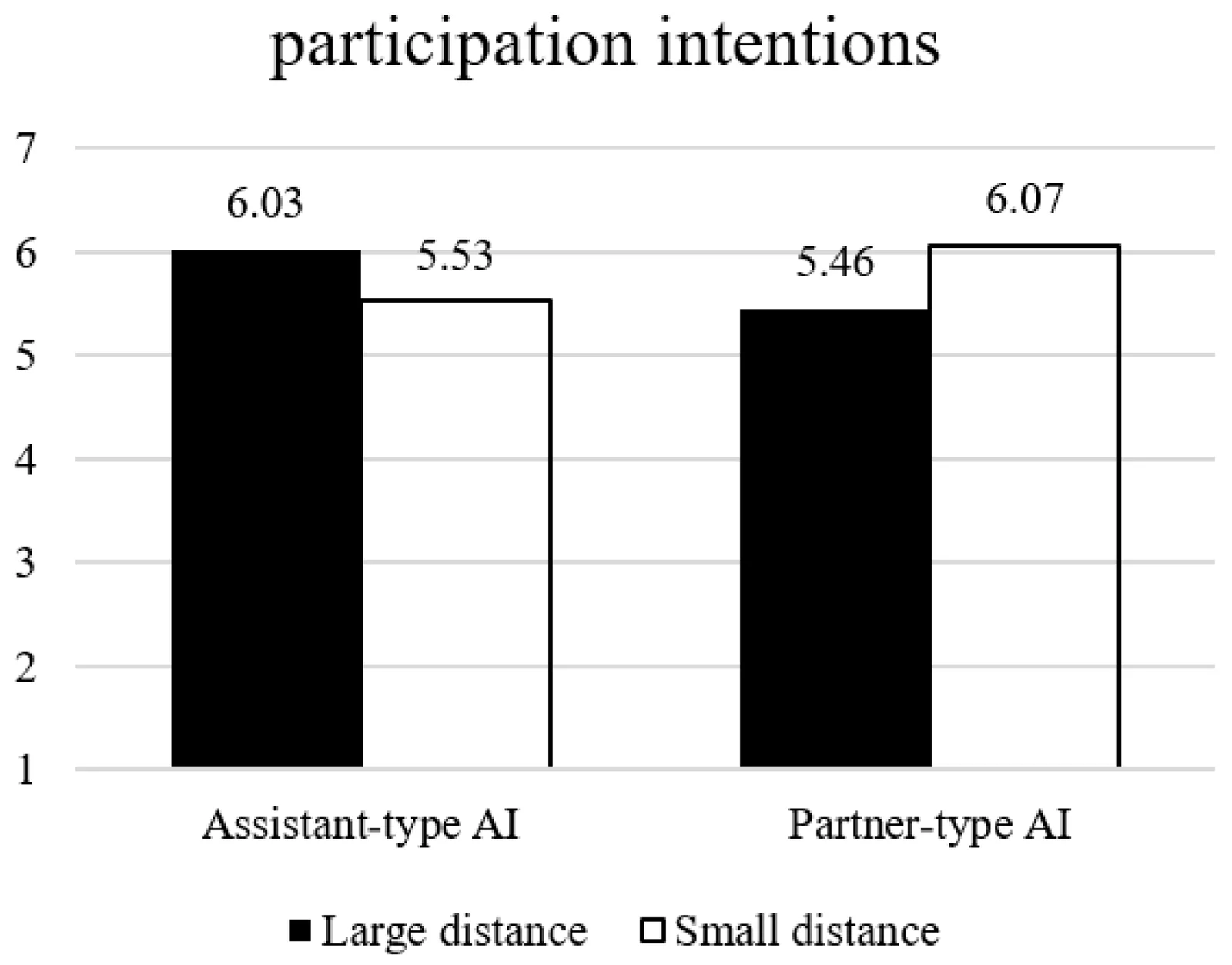
The interaction effect of AI relational roles and goal distance (community resource-circulation center initiative).

Using SPSS 26.0 and AMOS 21.0 to calculate, we found that at the 95% confidence interval level, the Cronbach’s α value for participation intention was 0.820, CR was 0.821, and AVE was 0.435; all factor loadings were greater than 0.5, indicating acceptable reliability and convergent validity of the scale. AI relational roles were coded as 1 = assistant-type AI and 2 = partner-type AI, and goal distance was coded as 1 = large-distance and 2 = small-distance. Study 1 adopted participation intention in the community resource-circulation center initiative as the dependent variable and conducted a two-way ANOVA analysis, finding a significant interaction effect between AI relational roles and goal distance (F(1, 247) = 53.62, *p* < 0.001, $\eta_{p}^{2}=0.178$, Table 1). Furthermore, independent-samples *t*-tests revealed that information emphasizing large-distance goals (vs. small-distance goals) from assistant-type AI more effectively stimulates participation intention (M_assistant-large_ = 6.03, SD = 0.45, M_assistant-small_ = 5.53, SD = 0.76, t(123) = 4.56, *p* < 0.001, Cohen’s d = 0.82, Figure 2). In contrast, the information provided by partner-type AI that emphasizes small-distance goals (vs. large-distance goals) is more effective in promoting participation intention (M_partner-large_ = 5.46, SD = 0.76, M_partner-small_ = 6.07, SD = 0.39, t(124) = −5.55, *p* < 0.001, Cohen’s d = 1.03, Figure 2). H1 was supported.

Because participants were randomly assigned to the experimental conditions, demographic variables were not used as control variables, including in the primary ANOVA. As a robustness check, we re-estimated the model while controlling for gender as a categorical variable (1 = male and 2 = female) and age as a continuous variable. The interaction between AI relational roles and goal distance remained significant (F(1, 245) = 51.83, *p* < 0.001, $\eta_{p}^{2}=0.175$). Thus, demographic variable adjustment did not alter the conclusion regarding H1.

## 4. Study 2: Collective Green Produce Subscription Program

### 4.1. Experimental Design

Study 2 was also conducted to test Hypothesis 1. We adopted a 2 (AI relational roles: assistant-type vs. partner-type) × 2 (goal distance: large vs. small) between-subjects experimental design.

In Study 2, we designed a collective green produce subscription program to measure the willingness of consumers in different groups to participate by setting different AI roles and goal distances. For the design of AI relational roles, we used a combination of images and text similar to that in Study 1 (see Appendix A). Similar to Study 1, Study 2 also established a goal for the implementation of collective behavior, namely, “the plan will be executed once 500 participants are reached.” By describing the current number of participants, we indicated the distance to the established goal, thereby stimulating participants’ perception and judgment of the goal’s proximity. We described the “large-distance goal” framing as follows: “The goal is to call for 500 consumers to participate, and now 102 people have already joined. As long as we reach 500 people, we will collectively order and deliver green organic agricultural products from farmers who use ecological farming methods.” We described the “small-distance goal” framing as follows: “The goal is to call for 500 consumers to participate, and now 398 people have already joined. As long as we reach 500 people, we will collectively order and deliver green organic agricultural products from farmers who use ecological planting methods.” We also designed a progress bar to visually present the relationship and distance between the number of participants and the goal number, allowing participants to understand it more intuitively (see Appendix A). Unlike Study 1, which presented goal distance information through text and numerical participant counts, Study 2 presented the numerical information together with a progress bar. The progress bar appeared in all four experimental conditions and was intended to make the completed and remaining proportions spatially visible in a format resembling an online collective campaign interface. By increasing the ease with which goal progress can be visualized, a nearly filled bar may increase the salience of goal proximity, attainability, and collective momentum, whereas a sparsely filled bar may make the remaining gap and the need for additional participation more salient (Cheema & Bagchi, 2011; Jin et al., 2023; van Teunenbroek et al., 2023; Habib et al., 2025). Therefore, the progress bar could be understood as a presentation feature of Study 2.

In order to test whether the experimental manipulation was successful, we specifically designed relevant test items. After viewing the stimulus material and answering questions related to participating in the collective green produce subscription program, participants needed to respond to the 7-point scale for manipulation check (1 = strongly disapprove, 7 = strongly approve). The four items for AI relational roles were the same as in Study 1. The four test items for goal distance were adapted from items in Study 1; by changing the wording of the program title, new test items were created. We tested the effectiveness of the manipulation by examining the differences in participants’ judgments and evaluations after being exposed to different materials in various experimental contexts. We adopted recruitment and data-quality procedures similar to those used in Study 1 to obtain a demographically diverse sample. At the same time, we did not allow participants to forward the questionnaire or answer it multiple times, ensuring the independence of the selected sample.

Participants in Study 2 were recruited through Credamo, a professional online research platform. After providing informed consent, participants were randomly assigned by the survey system with equal probability to one of the four experimental conditions. Questionnaire forwarding and multiple submissions were prohibited. A total of 260 responses were received. We sequentially excluded 15 responses because of patterned responses (such as ‘Z’-shaped options); the final analytic sample comprised 245 participants (N_female_ = 57.14%, M_age_ = 32.24, SD = 10.10).

### 4.2. Experimental Procedure

First, we designed a hypothetical scenario and guided the participants to imagine themselves in that scenario. We provided the following text: “Please imagine the following scenario: The online platform in your community has embedded an AI chatbot to improve service quality, which can recommend more targeted information to you and engage in daily conversations with you. Recently, the platform has been promoting knowledge related to sustainable behavior, and you saw the following information.” After that, participants were divided into 1/4 experimental conditions at random.

In the experiment, we also provided background information about the collective green produce subscription program to enhance realism. For example, we mentioned, “Current agricultural production is facing issues such as resource consumption, overuse of fertilizers and pesticides, and ecological and environmental pressures. Choosing green agricultural products not only relates to personal health but also helps support more sustainable agricultural production methods. We need to make joint efforts.” After reading the stimulus information, participants needed to respond to a 7-point scale regarding their participation intention in the collective green produce subscription program (1 = strongly disapprove, 7 = strongly approve). The scale includes six items, and its design is the same as in Study 1. Subsequently, participants answered the manipulation check items, including judgments on AI relational roles and goal distance. Finally, they answered questions related to demographic variables.

### 4.3. Results

We first examined whether the experimental manipulation was successful. The results of the paired-sample *t*-test showed that when participants receive information from an assistant-type AI, they are more likely to recognize the AI as an assistant rather than a partner (M_assistant_ = 6.09, SD = 0.64; M_partner_ = 3.05, SD = 1.96, *p* < 0.001); conversely, when they receive information from a partner-type AI, they are more likely to recognize the AI as a partner (M_assistant_ = 3.96, SD = 1.88; M_partner_ = 5.72, SD = 1.19, *p* < 0.001). Regarding the judgment of goal distance, when participants received information emphasizing large-distance goals, their ratings for the large dimension were higher (M_large_ = 5.60, SD = 0.90; M_small_ = 2.37, SD = 1.07, *p* < 0.001); conversely, when they received information emphasizing small-distance goals, their ratings for the small dimension were higher (M_large_ = 2.42, SD = 0.92; M_small_ = 5.80, SD = 0.74, *p* < 0.001). The above test results indicated that Study 2 successfully manipulated AI relational roles and goal distance.

In addition, manipulation effectiveness was assessed using independent-samples Welch’s *t*-tests comparing the relevant ratings across the corresponding assigned conditions. Participants assigned to the assistant-type AI condition rated the communicator as more assistant-like (M = 6.09, SD = 0.64) than participants assigned to the partner-type AI condition (M = 3.96, SD = 1.88; Welch’s t(146.88) = 11.78, *p* < 0.001, Cohen’s d = 1.52). Participants assigned to the partner-type AI condition rated the communicator as more partner-like (M = 5.72, SD = 1.19) than the participants assigned to the assistant-type AI condition (M = 3.05, SD = 1.96; Welch’s t(204.07) = 12.92, *p* < 0.001, Cohen’s d = 1.64). Regarding goal distance, participants assigned to the large-distance condition reported stronger large-distance perceptions (M = 5.60, SD = 0.90) than those assigned to the small-distance condition (M = 2.42, SD = 0.92; Welch’s t(242.29) = 27.31, *p* < 0.001, Cohen’s d = 3.49). Conversely, participants assigned to the small-distance condition reported stronger small-distance perceptions (M = 5.80, SD = 0.74) than those assigned to the large-distance condition (M = 2.37, SD = 1.07; Welch’s t(220.94) = 29.33, *p* < 0.001, Cohen’s d = 3.72). These results confirmed that the manipulations of AI relational roles and goal distance were successful.

**Figure 3:**
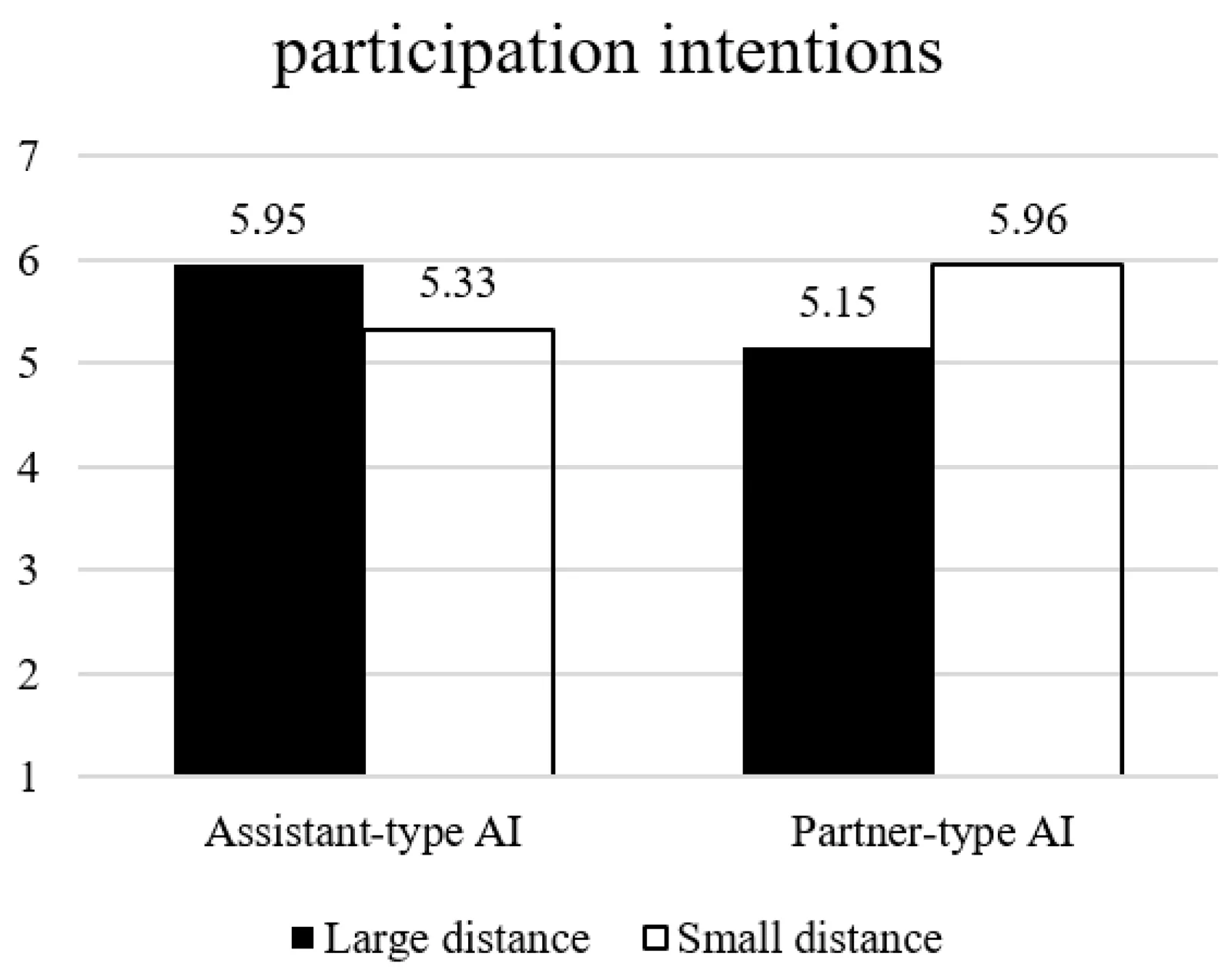
The interaction effect of AI relational roles and goal distance (collective green produce subscription).

Using SPSS 26.0 and AMOS 21.0 to calculate, we found that at the 95% confidence level, the Cronbach’s α value for participation intention was 0.903, CR was 0.904, AVE was 0.612, and all factor loadings were greater than 0.5, indicating good reliability and convergent validity of the scale. AI relational roles were coded as 1 = assistant-type AI and 2 = partner-type AI, and goal distance was coded as 1 = large-distance and 2 = small-distance. Study 2 adopted participation intention in the collective green produce subscription as the dependent variable and conducted a two-way ANOVA analysis, finding a significant interaction effect between AI relational roles and goal distance (F(1, 241) = 49.61, *p* < 0.001, $\eta_{p}^{2}=0.171$, Table 2). Furthermore, an independent-samples *t*-test revealed that information from assistant-type AI emphasizing large-distance goals (vs. small-distance goals) more effectively stimulates green produce subscription participation intention (M_assistant-large_ = 5.95, SD = 0.42, M_assistant-small_ = 5.33, SD = 0.95, t(122) = 4.64, *p* < 0.001, Cohen’s d = 0.85, Figure 3). In contrast, the information provided by partner-type AI emphasizing small-distance goals (vs. large-distance goals) is more effective in promoting participation intention (M_partner-large_ = 5.15, SD = 1.13, M_partner-small_ = 5.96, SD = 0.43, t(119) = −5.23, *p* < 0.001, Cohen’s d = 0.94, Figure 3). Once again, H1 was supported.

As in Study 1, we conducted a supplementary robustness analysis controlling for gender as a categorical variable (1 = male and 2 = female) and age as a continuous variable. The interaction between AI relational roles and goal distance remained significant (F(1, 239) = 46.70, *p* < 0.001, $\eta_{p}^{2}=0.163$), indicating that demographic adjustment did not alter the conclusion regarding H1.

From the elaboration likelihood model’s perspective, the progress bar may either reduce cognitive effort in interpreting numerical information or serve as a salient heuristic cue (Petty & Cacioppo, 1986; El Hedhli et al., 2023). In order to test this potential explanatory possibility, examining whether the progress bar used in Study 2 was accompanied by a broad change in response effort, we conducted supplementary analyses using total questionnaire completion time in seconds. Completion time is a paradata measure that can provide an indirect indicator of cognitive processing effort and processing speed (Schneider et al., 2023; Teckentrup et al., 2025). We used “Study” (Study 1 vs. Study 2) as the independent variable and response time as the dependent variable for analysis. A one-way ANOVA analysis showed no significant overall difference between Study 1 (M = 215.96, SD = 109.10) and Study 2 (M = 218.30, SD = 109.30; F(1, 494) = 0.06, *p* = 0.811). Separate one-way ANOVA analysis within the corresponding experimental conditions also revealed no significant study differences. Compared to the “assistant-large-Study 2” group, there was no significant difference in response time for “assistant-large-Study 1” (F(1, 130) = 0.022, *p* = 0.882). There was no significant difference between “assistant-small-Study 1” and “assistant-small-Study 2” (F(1, 115) = 0.028, *p* = 0.867). There was no significant difference between “partner-large-Study 1” and “partner-large-Study 2” (F(1, 117) = 0.132, *p* = 0.717). There was no significant difference between “partner-small-Study 1” and “partner-small-Study 2” (F(1, 126) = 0.021, *p* = 0.886). These results provided no evidence that the progress bar addition in Study 2 produced a significant change in total completion time, either overall or within a particular experimental condition.

## 5. Study 3: Collective Green-Packaging Commitment

### 5.1. Experimental Design

The purpose of Study 3 is to examine the interaction between AI relational roles and goal distance, as well as the mediating roles of identity affirmation and positive emotional resonance and their serial mediating role, specifically testing Hypotheses 1, 2, 3, and 4. We adopted a 2 × 2 factorial between-subjects experimental design.

In Study 3, we designed a collective green-packaging commitment to measure the willingness of consumers to participate. For the design of AI relational roles, we used a combination of images and text similar to that in Study 1 (see Appendix A). Study 3 also adopted different frameworks emphasizing “large” and “small” for the design of goal distance. We established a goal for the implementation of collective behavior, namely, “the plan will be initiated once 1000 participants are reached.” We indicated the distance to the stated goal by describing the current number of participants. We described the “large-distance goal” framing as follows: “The goal is to call for 1000 consumers to participate, and currently, 187 people have joined. As long as we reach 1000 people, we will contact the stores and supermarkets within the community to uniformly reduce the use of single-use plastic bags in future shopping, switching to paper or cloth bags for packaging.” We described the “small-distance goal” framing as follows: “The goal is to call for 1000 consumers to participate, and currently, 813 people have joined. As long as we reach 1000 people, we will contact the stores and supermarkets within the community to uniformly reduce the use of single-use plastic bags in future shopping, switching to paper or cloth bags for packaging.”

In order to test whether the experimental manipulation was successful, we designed test items for evaluation. After viewing the stimulus material and answering questions related to participating in the collective green-packaging commitment, participants needed to respond to the 7-point scale for manipulation check (1 = strongly disapprove, 7 = strongly approve). The four items for AI relational roles were the same as in Study 1. The four measurement items for goal distance were adapted from items in Study 1; by changing the wording of the program title, new test items were created. We tested the differences in participants’ evaluations after receiving material stimuli in different experimental contexts to verify the effectiveness of the manipulation. We adopted recruitment and data-quality procedures similar to those used in Studies 1 and 2 to obtain a demographically diverse sample. Simultaneously, we did not allow participants to forward the questionnaire or answer it multiple times, ensuring the independence of the selected sample.

Similarly, participants in Study 3 were recruited through Credamo, a professional online research platform. After providing informed consent, participants were randomly assigned by the survey system with equal probability to one of the four experimental conditions. Questionnaire forwarding and multiple submissions were prohibited. A total of 300 responses were received. We sequentially excluded 16 responses because of patterned responses (such as ‘Z’-shaped options); the final analytic sample comprised 284 participants (N_female_ = 63.03%, M_age_ = 31.85, SD = 10.23).

### 5.2. Experimental Procedure

First, we designed a hypothetical scenario and informed the participants, “Please imagine the following scenario: The online platform in your community has embedded an AI chatbot to improve service quality, which can recommend more targeted information to you and engage in daily conversations with you. Recently, the platform has been promoting knowledge related to sustainable behavior, and you saw the following information.” After that, participants were divided into 1/4 experimental conditions at random.

In this formal experiment, we also provided background information on the collective green-packaging commitment to enhance realism. For example, we mentioned, “We are facing a severe plastic packaging pollution problem. The extensive use of single-use plastic bags will increase the environmental burden and have long-term impacts on the ecosystem. In order to promote more sustainable consumption practices, we need to make joint efforts.” After reading the stimulus information, participants needed to respond to a 7-point scale regarding their participation intention in the collective green-packaging commitment (1 = strongly disapprove, 7 = strongly approve). The scale design is the same as in Study 1. Next, participants also needed to answer the 7-point scale for identity affirmation and positive emotional resonance. The items for identity affirmation include (1) “I regard myself as an individual concerned with environmental issues”; (2) “I regard myself as a sustainable and green person”; (3) “Engaging in the above program instills a sense of identity as a green and sustainable person”; and (4) “Should I participate in the above program, I would experience complete self-satisfaction” (adapted from Confente et al., 2020). The items for positive emotional resonance include (1) “The message above stirred my emotions”; (2) “I felt refreshed when I saw the message above”; (3) “I felt comfortable and relaxed when I saw the message above”; (4) “I felt satisfied and at ease when I saw the message above”; and (5) “I felt happy when I saw the message above” (adapted from Jang & Namkung, 2009). These five items were used to assess participants’ general positive emotional resonance to the message. We used the term “positive emotional resonance” because the items capture positively valenced affective states rather than discrete moral or social emotions. Subsequently, participants answered the manipulation check items, including judgments on AI relational roles and goal distance. Finally, they answered questions related to demographic variables.

### 5.3. Results

First, we examined whether the experimental manipulation was successful. The results of the paired-sample *t*-test showed that when participants receive information from an assistant-type AI, they are more likely to recognize the AI as an assistant rather than a partner (M_assistant_ = 5.94, SD = 0.77; M_partner_ = 3.69, SD = 1.96, *p* < 0.001); conversely, when they receive information from a partner-type AI, they are more likely to recognize the AI as a partner (M_assistant_ = 4.20, SD = 1.89; M_partner_ = 5.64, SD = 1.28, *p* < 0.001). Regarding the judgment of goal distance, when participants received information emphasizing large-distance goals, their ratings for the large dimension were higher (M_large_ = 5.79, SD = 0.92; M_small_ = 2.30, SD = 1.17, *p* < 0.001); conversely, when they received information emphasizing small-distance goals, their ratings for the small dimension were higher (M_large_ = 2.35, SD = 1.06; M_small_ = 5.90, SD = 0.80, *p* < 0.001). The above test results indicated that Study 3 successfully manipulated AI relational roles and goal distance.

In addition, manipulation effectiveness was supplemented using independent-samples Welch’s *t*-tests comparing the relevant ratings across the corresponding assigned conditions. Participants assigned to the assistant-type AI condition rated the communicator as more assistant-like (M = 5.94, SD = 0.77) than participants assigned to the partner-type AI condition (M = 4.20, SD = 1.88; Welch’s t(194.48) = 10.32, *p* < 0.001, Cohen’s d = 1.20). Participants assigned to the partner-type AI condition rated the communicator as more partner-like (M = 5.64, SD = 1.28) than participants assigned to the assistant-type AI condition (M = 3.69, SD = 1.96; Welch’s t(233.60) = 9.87, *p* < 0.001, Cohen’s d = 1.18). Regarding goal distance, participants assigned to the large-distance condition reported stronger large-distance perceptions (M = 5.79, SD = 0.92) than those assigned to the small-distance condition (M = 2.35, SD = 1.06; Welch’s t(277.18) = 29.26, *p* < 0.001, Cohen’s d = 3.47). Conversely, participants assigned to the small-distance condition reported stronger small-distance perceptions (M = 5.90, SD = 0.80) than those assigned to the large-distance condition (M = 2.30, SD = 1.17; Welch’s t(246.40) = 30.18, *p* < 0.001, Cohen’s d = 3.59). These results confirmed that the manipulations of AI relational roles and goal distance were successful.

Using SPSS 26.0 and AMOS 21.0 to calculate, we found that at the 95% confidence interval level, the Cronbach’s α values for participation intention, identity affirmation, and positive emotional resonance were 0.896, 0.812, and 0.862, respectively; CR values were 0.897, 0.811, and 0.866, respectively; and AVE values were 0.594, 0.518, and 0.564, respectively; all factor loadings were greater than 0.5, indicating good reliability and convergent validity of the scales. To assess discriminant validity, we compared the hypothesized three-factor CFA model with three two-factor alternatives and a single-factor model, namely two-factor model 1 (combining participation intention and identity affirmation), two-factor model 2 (combining participation intention and positive emotional resonance), two-factor model 3 (combining identity affirmation and positive emotional resonance), and the single-factor model (all measurement items loading onto the same latent variable). The comparison found that the three-factor model had better goodness of fit (χ^2^/df = 2.04, CFI = 0.965, TLI = 0.957, RMSEA = 0.061, SRMR = 0.038) and was significantly superior to the two-factor model 1 (Δχ^2^ = 16.53, *p* < 0.001), two-factor model 2 (Δχ^2^ = 45.13, *p* < 0.001), two-factor model 3 (Δχ^2^ = 24.12, *p* < 0.001), and the single-factor model (Δχ^2^ = 55.20, *p* < 0.001). Therefore, when any two constructs or all three constructs were combined, the model fit significantly decreased. This result provided CFA model comparison evidence for the empirical distinctiveness of the three constructs.

**Figure 4:**
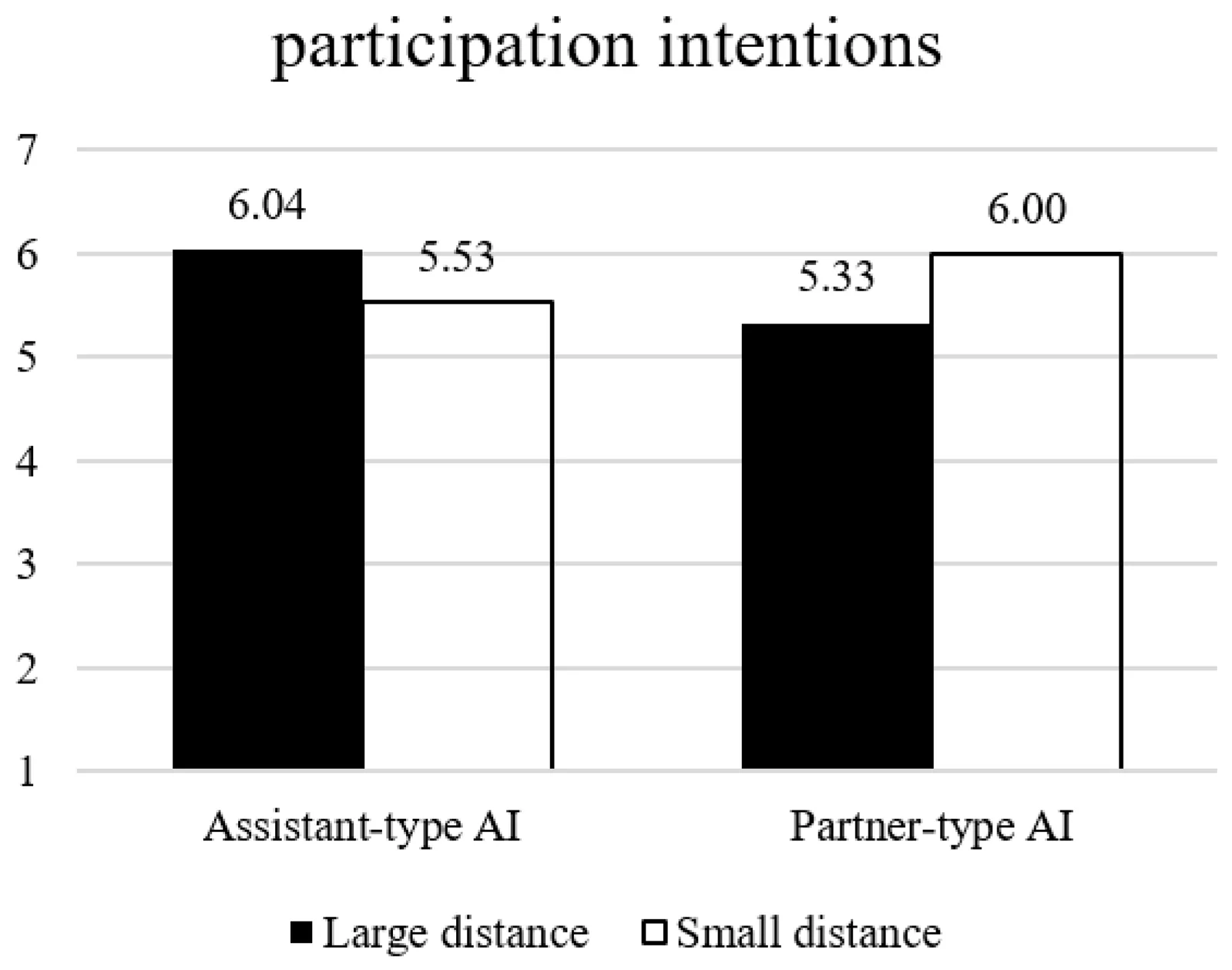
The interaction effect of AI relational roles and goal distance (collective green-packaging commitment).

AI relational roles were coded as 1 = assistant-type AI and 2 = partner-type AI, and goal distance was coded as 1 = large-distance and 2 = small-distance. Study 3 adopted participation intention in the collective green-packaging commitment as the dependent variable and conducted a two-way ANOVA analysis, finding a significant interaction effect between AI relational roles and goal distance (F(1, 280) = 46.29, *p* < 0.001, $\eta_{p}^{2}=0.142$, Table 3). Furthermore, an independent-samples *t*-test revealed that information emphasizing large-distance goals (vs. small-distance goals) from an assistant-type AI more effectively stimulates green-packaging commitment participation intention (M_assistant-large_ = 6.04, SD = 0.59, M_assistant-small_ = 5.53, SD = 0.75, t(136) = 4.48, *p* < 0.001, Cohen’s d = 0.76, Figure 4). In contrast, the information provided by partner-type AI that emphasizes small-distance goals (vs. large-distance goals) is more effective in promoting participation intention (M_partner-large_ = 5.33, SD = 1.02, M_partner-small_ = 6.00, SD = 0.46, t(144) = −5.07, *p* < 0.001, Cohen’s d = 0.84, Figure 4). H1 was supported again.

Consistent with Studies 1 and 2, when gender as a categorical variable (1 = male and 2 = female) and age as a continuous variable were included in a robustness analysis, the interaction between AI relational roles and goal distance remained significant (F(1, 278) = 41.05, *p* < 0.001, $\eta_{p}^{2}=0.129$). The conclusion regarding H1 therefore remained unchanged.

We then examined the mediating role of identity affirmation. Using the PROCESS plugin (Version 4.0) for Bootstrap analysis, Model 8 was selected 5000 bootstrap resamples and a 95% confidence interval. AI relational roles were coded as 1 = assistant-type AI and 2 = partner-type AI, and goal distance was coded as 1 = large-distance and 2 = small-distance. The results showed that the interaction item of AI relational roles and goal distance positively influenced participation intention (β = 0.54, t = 4.76, SE = 0.11, CI = [0.32, 0.76], *p* < 0.001) and identity affirmation (β = 0.78, t = 4.74, SE = 0.17, CI = [0.46, 1.11], *p* < 0.001). The positive impact of identity affirmation on participation intention was also significant (β = 0.82, t = 20.77, SE = 0.04, CI = [0.74, 0.89], *p* < 0.001). The index of moderated mediation for the indirect effect through identity affirmation in Model 8 was 0.641 (BootSE = 0.159, 95% bootstrap CI = [0.346, 0.966]). Comprehensive findings are available in Table 4, which illustrates the identity affirmation’s mediating function. Further analysis across different goal distances revealed that in the large-distance goal group, identity affirmation had a significant mediating effect on the impact of AI relational roles on participation intention (CI = [−0.57, −0.12], not including 0); in the small-distance goal group, the mediating effect still existed (CI = [0.14, 0.49], not including 0) (Table 5), thus validating H2.

In addition, Study 3 examined the mediating role of positive emotional resonance. Using the PROCESS plugin (Version 4.0) for Bootstrap analysis, Model 8 selected 5000 bootstrap resamples under a 95% confidence interval. AI relational roles were coded as 1 = assistant-type AI and 2 = partner-type AI, and goal distance was coded as 1 = large-distance and 2 = small-distance. The results showed that the interaction item positively influenced participation intention (β = 0.49, t = 4.35, SE = 0.11, CI = [0.27, 0.71], *p* < 0.001) and positive emotional resonance (β = 0.98, t = 5.07, SE = 0.19, CI = [0.60, 1.37], *p* < 0.001). The positive impact of positive emotional resonance on participation intention was also significant (β = 0.70, t = 21.14, SE = 0.03, CI = [0.64, 0.77], *p* < 0.001). The index of moderated mediation for the indirect effect through positive emotional resonance was 0.690 (BootSE = 0.162, 95% bootstrap CI = [0.391, 1.028]). Comprehensive findings are available in Table 6, which demonstrated the positive emotional resonance’s mediating function. Further analysis under different goal distances revealed that in the large-distance goal group, positive emotional resonance had a significant mediating effect on the impact of AI relational roles on participation intention (CI = [−0.59, −0.14], not including 0); in the small-distance goal group, the mediating effect remained significant (CI = [0.16, 0.55], not including 0) (Table 7), thus validating H3.

**Figure 5:**
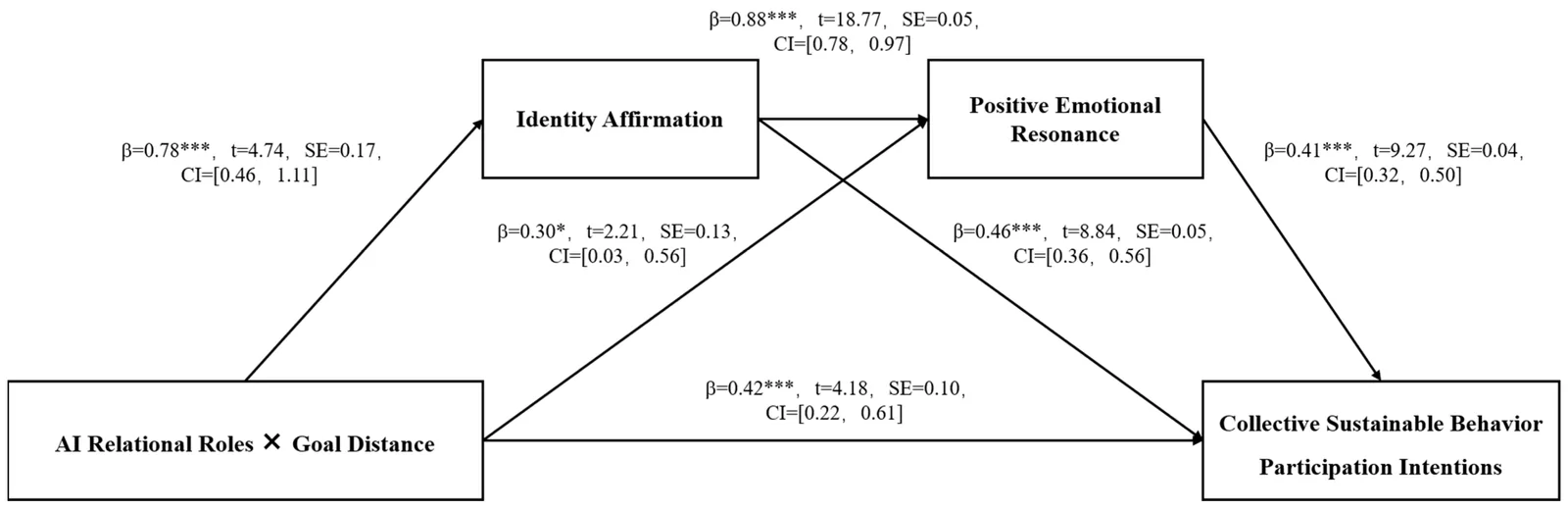
Serial mediating role of identity affirmation and positive emotional resonance. Note: * *p* < 0.05, *** *p* < 0.001.

Study 3 further examined the serial mediating effect of identity affirmation and positive emotional resonance. Using the PROCESS plugin (Version 4.0) for Bootstrap analysis, Model 85 selected 5000 bootstrap resamples under a 95% confidence interval. AI relational roles were coded as 1 = assistant-type AI and 2 = partner-type AI, and goal distance was coded as 1 = large-distance and 2 = small-distance. The results revealed that the interaction item positively influenced participation intention (β = 0.42, t = 4.18, SE = 0.10, CI = [0.22, 0.61], *p* < 0.001), identity affirmation (β = 0.78, t = 4.74, SE = 0.17, CI = [0.46, 1.11], *p* < 0.001), and positive emotional resonance (β = 0.30, t = 2.21, SE = 0.13, CI = [0.03, 0.56], *p* = 0.028). The impact of identity affirmation on participation intention was significant (β = 0.46, t = 8.84, SE = 0.05, CI = [0.36, 0.56], *p* < 0.001). The positive impact of positive emotional resonance on participation intention was also significant (β = 0.41, t = 9.27, SE = 0.04, CI = [0.32, 0.50], *p* < 0.001). The positive impact of identity affirmation on positive emotional resonance was of similar significance (β = 0.88, t = 18.77, SE = 0.05, CI = [0.78, 0.97], *p* < 0.001). The index of moderated mediation for the focal serial indirect path through identity affirmation and positive emotional resonance in Model 85 was 0.281 (BootSE = 0.079, 95% bootstrap CI = [0.145, 0.454]). This revealed the serial mediating effect of identity affirmation and positive emotional resonance (Figure 5). Further analysis of the serial mediating role across different goal distances revealed that, in the large-distance goal group, identity affirmation and positive emotional resonance exerted a significant serial mediating effect on the influence of AI relational roles on participation intention (CI = [−0.26, −0.06], not including 0); in the small-distance goal group, the serial mediating effect remained significant (CI = [0.05, 0.24], not including 0; Table 8), thus validating H4.

The significant indirect effect was statistically consistent with the hypothesized ordering through identity affirmation and positive emotional resonance, thereby providing support for H4. However, the coefficients should not be interpreted as demonstrating that the three psychological responses occurred in an empirically established temporal causal sequence. Identity affirmation, positive emotional resonance, and participation intention were assessed within the same experimental session. Therefore, the analysis evaluated a theory-guided pattern of indirect associations rather than directly observing the temporal development of the proposed psychological process.

To evaluate the robustness of the mediation results, we re-estimated Models 8 and 85 with gender as a categorical variable (1 = male and 2 = female) and age as a continuous variable and used 5000 bootstrap resamples. The conditional indirect effect through identity affirmation remained significant under both the large-distance goal condition (β = −0.303, 95% bootstrap CI = [−0.524, −0.102], excluding 0) and the small-distance goal condition (β = 0.285, 95% bootstrap CI = [0.111, 0.480], excluding 0). The conditional indirect effect through positive emotional resonance also remained significant under the large-distance goal condition (β = −0.329, 95% bootstrap CI = [−0.556, −0.125], excluding 0) and the small-distance goal condition (β = 0.323, 95% bootstrap CI = [0.149, 0.536], excluding 0). Finally, the serial indirect effect through identity affirmation and positive emotional resonance remained significant under both the large-distance goal condition (β = −0.133, 95% bootstrap CI = [−0.232, −0.045], excluding 0) and the small-distance goal condition (β = 0.125, 95% bootstrap CI = [0.045, 0.231], excluding 0). Thus, controlling for gender and age did not alter the conclusions regarding H2–H4.

## 6. General Discussion

This work focuses on the impact mechanism of AI relational roles and goal distance on participation intentions in collective sustainable behavior. Across three experimental studies involving different collective sustainable initiatives, the two AI role conditions, as operationalized in this research, consistently interact with goal distance in predicting participation intention. Therefore, the findings provide evidence regarding the comparative effects of assistant-type and partner-type communication packages used in the experiments. This finding is consistent with the conclusions of Jin et al. (2023) regarding collective goals. They pointed out that when the goal distance is large, consumers are more likely to enter a thinking-oriented state, focusing more on whether the goal is feasible and how to achieve it. Conversely, when the collective goal distance is small, consumers are more likely to enter a feeling-oriented state, paying more attention to emotional values and the immediate significance of participation. Therefore, large-distance goals are more suitable to be conveyed by assistant-type AI with planning and goal management characteristics, while small-distance goals are more suitable to be stimulated by partner-type AI that emphasizes companionship and immediate interaction to encourage participation. This also responds to the viewpoint of Hertel et al. (2026) regarding sustainable development issues, which states that different entities need to jointly create conditions for action. In other words, collective sustainable behavior not only depends on the collective goal itself but also on the interaction between the action entities and the information cues. At the same time, this work also resonates with the research by Chi et al. (2024) on AI anthropomorphism, which points out that the competence and warmth in anthropomorphic robots can enhance consumer preference and attachment. In this work, assistant-type AI is more aligned with competence-oriented AI, and partner-type AI is more aligned with warmth-oriented AI, thereby extending the fit effect from AI internal features to the interaction between AI relational roles and goal distance. In this research, the presentation of goal distance information also differed across Studies 1 and 2. Study 1 required participants to infer goal progress from numerical counts, whereas Study 2 additionally included a progress bar. A nearly filled bar could increase the salience of proximity and attainability, while a sparsely filled bar may highlight the remaining gap and need for support (Cheema & Bagchi, 2011; Y. Kim & Reeck, 2019; van Teunenbroek et al., 2023; Habib et al., 2025). From the elaboration likelihood model perspective, the progress bar may either reduce cognitive effort in interpreting numerical information or serve as a salient heuristic cue (Petty & Cacioppo, 1986; El Hedhli et al., 2023). In order to test this potential explanatory possibility, supplementary analysis of completion time was conducted. The results showed no overall difference between studies or within any condition, suggesting no significant change in processing time. However, as completion time is an indirect measure and the studies differ in context, these results do not rule out more specific effects on attention, fluency, or interpretation. Study 2 should therefore be viewed as a conceptual replication in a more visual, platform-like setting rather than a direct test of the progress bar’s independent effect.

However, our findings also differ from some studies on goal proximity. Some research suggests that small-distance goals are often more motivating (Kivetz et al., 2006; Liao et al., 2025), whereas this work finds that goal distance can match with AI relational roles and thus work together; neither AI relational roles nor goal distance has a universally superior persuasive effect. Instead, their effectiveness depends on whether the support mode represented by the AI role corresponds to the psychological requirements created by the current stage of collective goal pursuit. The reason for this difference may lie in the fact that existing research often focuses on individual reward goals or loyalty programs, whereas collective sustainable behavior has public, other-benefit, and interdependent attributes. Consumers cannot independently decide whether the goal can be achieved, so their willingness to participate is also shaped by the persuader’s characteristics, the extent of their own contribution, and the level of others’ efforts.

It is worth noting that collective sustainable behavior is embedded in a social environment in which consumers infer what others are doing, what the group approves of, and whether their participation aligns with shared expectations. In this research, the number of previous participants has conveyed goal distance but may also convey descriptive-norm information, functioning as social proof and a signal of project support and attainability (Schultz et al., 2007; Y. Kim & Reeck, 2019; van Teunenbroek et al., 2023). Prior research shows that descriptive norms can strongly shape pro-environmental and prosocial behavior by signaling behavioral appropriateness and reducing uncertainty in collective contexts (Nolan et al., 2008; Goldstein et al., 2008). AI relational roles may shape how these social cues are interpreted. A partner-type AI, through inclusive and companion-like communication, may enhance feelings of group belonging and shared purpose, making participation feel more like a collective endeavor and strengthening social identity (Ahn et al., 2014). In contrast, an assistant-type AI may translate collective expectations into clearer behavioral standards and procedural guidance, increasing the salience of injunctive norms and perceived behavioral clarity (Cialdini et al., 1990; Ajzen, 1991). Thus, partner-type AI may emphasize relational and belonging-related norms, whereas assistant-type AI may emphasize informational and coordination-related norms. These perspectives extend AI relational roles beyond identity and emotion to the broader social context of collective action. These normative pathways should be examined in further research to clarify how AI roles shape consumers’ interpretation of collective social information.

Furthermore, we found that identity affirmation and positive emotional resonance each play a mediating role, and there is a serial mediating effect. This is highly consistent with the findings in the research on sustainable behavior regarding self-identity and emotional connection (Dermody et al., 2018; Kao, 2019). Becerra et al. (2023) pointed out that green self-identity is an important psychological foundation for green behavior intention, even more so than some product value variables in explaining green behavior and recommendation intention; Yu et al. (2024) found that emotional perception can significantly influence sustainable purchase intention. This work further identifies a positive association between sustainable identity affirmation and positive emotional resonance, indicating that the two constructs jointly account for part of the relationship between the interaction of AI relational roles and goal distance and participation intention. The proposed ordering is theoretically plausible because perceiving collective sustainable action as self-congruent may enhance its personal value and emotional appeal, with identity-related appraisal potentially fostering more positive emotional responses that are associated with stronger participation intention. This provides the conceptual rationale for the serial model within the limits of the data. It is worth noting that the serial indirect effect should be interpreted as a statistical pattern consistent with the proposed ordering rather than evidence of a temporal causal sequence.

## 7. Conclusions

### 7.1. Research Findings

This work across three experimental studies involving different collective sustainable initiatives consistently reveals the relational role of AI (assistant-type vs. partner-type) and goal distance (large vs. small) in influencing participation intention in collective sustainable behaviors. Additionally, we verified the mediation effect of identity affirmation and positive emotional resonance, as well as their serial mediating role. The study found the following: (1) There is an interaction between AI relational roles and goal distance. Assistant-type AI (vs. partner-type AI) is more effective in conveying large-distance goals to enhance participation intention in collective sustainable behaviors, while partner-type AI (vs. assistant-type AI) performs better when combined with small-distance goals. (2) Identity affirmation and positive emotional resonance each mediate the interaction effect. (3) Identity affirmation and positive emotional resonance play a serial mediating role. The serial indirect-effect pattern is consistent with an ordered association in which stronger sustainable identity affirmation is related to stronger positive emotional resonance, which is associated with greater participation intention.

### 7.2. Theoretical Contributions

First, this work expands the theoretical boundaries of the Computers Are Social Actors (CASA) and AI-mediated communication (AIMC) frameworks, revealing that AI relational roles influence consumers’ collective sustainable behavior, depending on whether the social characters it portrays match the goal framing. Existing research has mostly focused on the differences in the impact of AI versus human information sources or emphasized that relationality can enhance AI’s social presence, trust, and persuasive effects (Blut et al., 2021). This work distinguishes AI relational roles into assistants and partners, finding that the two are suitable for different goal distances, further distinguishing the differences in relational expectations and communication functions activated by different AI relational roles. Assistant-type AI, owing to its guiding and normative characteristics, is more suitable for conveying large-distance goals that emphasize long-term value and future vision; on the other hand, partner-type AI, with its characteristics of companionship and joint participation, is more suitable for conveying small-distance goals that emphasize immediate action and psychological closeness. This finding advances AI persuasion research from the static comparison of information source attributes to the dynamic matching of “AI role-goal framing,” further refining the understanding of the differential roles of AI in influencing sustainable behavior. This provides a new theoretical perspective for understanding the mechanisms by which AI relational roles influence the socialization process of consumer behavior.

Second, this research advances regulatory fit theory by identifying a relational and dynamically changing form of fit in AI-mediated collective goal pursuit. Regulatory fit theory traditionally explains how congruence between individuals’ goal orientations or concerns and their manner of goal pursuit creates stronger engagement and a sense of “feeling right,” thereby increasing perceived value and persuasion (Higgins, 2000; Avnet & Higgins, 2006; Cesario et al., 2004). Most applications emphasize the fit between consumers’ regulatory orientations and message framing, decision strategies, or goal-pursuit means. The present research extends the unit of fit to a communicator role and goal-state relationship. Specifically, AI relational roles influence how support is socially delivered: assistant-type AI embodies a cognitive and instrumental mode of engagement, whereas partner-type AI embodies an affective and relational mode. Goal distance, meanwhile, dynamically changes the psychological concerns that consumers bring to a collective goal. It demonstrates that the regulatory fit can emerge from the alignment between a social communicator’s relational role and the changing stage of collective goal pursuit. As a collective campaign progresses and goal distance decreases, the AI role that provides the stronger fit may change accordingly. Thus, the regulatory fit is not only a property of how a message is framed but can also be shaped by who the AI is perceived to be and when the communication occurs during goal pursuit. This contribution also introduces a dynamic dimension to the regulatory fit.

Third, this work further extends the identity-value model to the field of sustainable behavior, revealing AI-driven collective sustainable behavior through a chain of psychological pathways involving identity affirmation and positive emotional resonance. Existing research on the impact of AI persuasion on sustainable behavior often emphasizes AI’s professionalism and accuracy, with less focus on how to activate individuals’ identity perception and emotional connection in communication. We identified and validated the serial mediating effect of sustainable identity affirmation and positive emotional resonance, indicating that in sustainable issues with attributes and public interest orientation, AI can influence consumers by designing relational roles that evoke identity responses and positive emotional resonance similar to interpersonal interactions. Thus, the research extends the identity-value model by showing how identity-based motivation can develop into an affective connection with a collective goal. This moves the model beyond an exclusively individual self-regulation context and demonstrates its relevance to socially embedded, AI-mediated, and collectively oriented sustainable action, thereby deepening the understanding of the micro-mechanisms of how AI can promote sustainable behavior.

### 7.3. Practical Implications

The primary practical audience for this research comprises sustainability marketers, campaign managers, and digital engagement managers in companies, brands, and online platforms that use AI-enabled communication to recruit consumers into collective environmental initiatives. These professionals are involved in shaping decisions regarding the relational role assigned to an AI communicator, the type of support it provides, and whether its communication strategy should change as a collective goal progresses. The findings therefore offer insights into the design and management of consumer-facing sustainability campaigns. The study found that there is an interaction effect between AI relational roles and goal distance: Assistant-type AI combined with large-distance goals is more effective in enhancing consumers’ participation intention, while partner-type AI combined with small-distance goals is more effective. Therefore, when designing sustainable behavior initiatives, marketers should not simply pursue emotionalization of AI but should choose the appropriate relational roles based on goal distance. When the behavior goal is long-term and macro, such as long-term carbon reduction plans, ecological governance, and the formation of green lifestyles, an assistant-type AI is more suitable as the communication subject. Through professional advice, action planning, and goal explanation, it helps consumers understand the long-term value of collective behaviors (Y. Chen & Prentice, 2025). Conversely, when the behavior goals are more specific, immediate, and easy to execute, such as time-limited environmental activities, donation initiatives, garbage classification check-ins, or short-term green actions, partner-type AI can better lower participation thresholds and enhance consumers’ motivation to act through companion-like interactions and encouraging language.

Secondly, marketers should manage the fit between AI relational roles and goal distance dynamically throughout the lifecycle of a collective sustainability campaign. Large and small goal distances should not be regarded only as fixed campaign characteristics; they may also represent changing stages as a collective campaign progresses. When the goal is far from completion, marketers can rely primarily on assistant-type AI and provide action plans, factual evidence, and information about the long-term value and feasibility of participation. As accumulated contributions reduce the remaining goal distance, the communication strategy can gradually shift toward partner-type interaction by highlighting shared momentum, goal attainability, and the experience of completing the goal together. Campaign systems can therefore use progress-based triggers to adjust conversational scripts and communication framing. Because perceived goal distance may differ from objective progress, marketers should also communicate both the progress already achieved and the contribution still required.

Thirdly, marketers should attend to the roles of sustainable identity affirmation and positive emotional resonance in participation-related communication. When communicating sustainable actions that highlight environmental values and identity expression, marketers can reinforce consumers’ sustainable identity affirmation through green identity labels, long-term contribution records, sustainable achievement badges, and community rankings. These communication elements may help consumers interpret their participation in collective sustainable behavior as an affirmation of “I am a person who cares about sustainable development and is willing to take collective action.” At the same time, communication strategies can incorporate warm narratives, collective-participation discourse, immediate encouragement, and emotional feedback to elicit positive emotional resonance and convey the significance of individual actions and collective value. Because the serial indirect effect pattern is consistent with ordered associations among identity affirmation, positive emotional resonance, and participation intention, practitioners may coordinate identity-relevant and affective communication elements. This implication should be understood as a theory-informed design implication rather than evidence that identity affirmation temporally causes positive emotional resonance or that positive emotional resonance subsequently causes participation intention.

### 7.4. Limitations and Future Research

This research still has certain limitations. First, this work categorizes AI relational roles into assistant and partner types to isolate two modes of support. Real-world AI personas can be hybrid and dynamic, including mentors, coaches, community facilitators, or advisors that combine instrumental guidance with empathy and encouragement (Ricon, 2025). Such roles may not lie on a simple binary continuum. A coach, for example, may strengthen sustainable identity through goal setting while simultaneously eliciting positive emotional resonance through encouragement; a mentor may shift between these functions as goal progress changes. Future research should represent AI roles along separable dimensions of instrumental guidance and relational–emotional support, test hybrid profiles and role transitions, examine whether they produce additive, interactive, or stage-dependent effects on identity affirmation, positive emotional resonance, and participation intention, and continue comparing humans and neutral AI. In addition, the present manipulation varied the role label, self-description, and communication style as a composite package. Consequently, the present design makes it difficult to distinguish the individual effects of these factors. The stimuli also consisted of static, one-shot AI messages rather than reciprocal, multi-turn relational interaction. Future research should manipulate role labels, warmth, competence, relational closeness, and communication style independently and should compare static messages with dynamic conversational AI interactions, and observe and measure actual behavior rather than intentions. Second, drawing on the research by Jin et al. (2023), goal distance was operationalized by varying the number of existing participants relative to a fixed participation target in this research. Although this manipulation successfully altered perceived goal proximity, the larger participant count in the small-distance condition may also have conveyed stronger descriptive norms or social proof, namely, that many other people had already chosen to participate (Cialdini et al., 1990). Future research can attempt other manipulation and design methods for goal distance by manipulating and distinguishing tests with existing participation numbers, only presenting the remaining proportion without showing participation numbers, or directly measuring perceived descriptive norms or social proof to further distinguish and analyze their mechanisms of action. Third, although robustness checks including available demographic covariates show that the interaction effect remains stable, the studies do not measure pre-treatment environmental knowledge, prior participation in environmental initiatives, environmental involvement, or environmental skepticism, all of which may shape reliance on instrumental (assistant-type AI) versus relational (partner-type AI) support. Therefore, future research should measure them prior to exposure using validated scales and test their moderating roles. Fourth, the use of an identical image across AI role conditions isolates role-related language, but real digital assistants and partners may also signal their roles through attire, facial expression, posture, or other visual cues. Future research could manipulate textual relational roles and visual role identities independently to distinguish the separate and interactive effects of verbal and visual role cues. Such studies should also pretest visual attractiveness, human likeness, avatar familiarity, avatar formality, and color preference to ensure that visual-role effects are not attributable to unintended differences between images. Fifth, although the serial mediation analysis is consistent with the proposed ordering of identity affirmation, positive emotional resonance, and participation intention, the present design cannot establish their temporal causal sequence. All variables were measured within a single experimental session. Thus, the analysis cannot determine whether identity affirmation precedes positive emotional resonance. Future research should use temporally separated measurements, longitudinal designs, or experimental manipulations to better test the ordering and compare alternative mediation models.

## Figures and Tables

**Table 1:** ANOVA results of Study 1.

Source	Type III Sum of Squares	DF	Mean Square	F Value	*p* Value	Partial Eta Squared
Intercept	8298.583	1	8298.583	22,983.894	<0.001	0.989
AI Relational Roles	0.010	1	0.010	0.028	0.868	<0.001
Goal Distance	0.201	1	0.201	0.556	0.457	0.002
AI × Goal	19.360	1	19.360	53.619	<0.001	0.178
Error	89.182	247	0.361			
Total	8533.472	251				
Corrected Total	108.768	250				

**Table 2:** ANOVA results of Study 2.

Source	Type III Sum of Squares	DF	Mean Square	F Value	*p* Value	Partial Eta Squared
Intercept	7676.354	1	7676.354	12,159.771	<0.001	0.981
AI Relational Roles	0.469	1	0.469	0.743	0.390	0.003
Goal Distance	0.558	1	0.558	0.885	0.348	0.004
AI × Goal	31.318	1	31.318	49.610	<0.001	0.171
Error	152.141	241	0.631			
Total	7877.083	245				
Corrected Total	184.547	244				

**Table 3:** ANOVA results of Study 3.

Source	Type III Sum of Squares	DF	Mean Square	F Value	*p* Value	Partial Eta Squared
Intercept	9301.127	1	9301.127	17,443.274	<0.001	0.984
AI Relational Roles	1.053	1	1.053	1.975	0.161	0.007
Goal Distance	0.443	1	0.443	0.830	0.363	0.003
AI × Goal	24.684	1	24.684	46.293	<0.001	0.142
Error	149.302	280	0.533			
Total	9504.111	284				
Corrected Total	175.594	283				

**Table 4:** Results of the mediating analysis regarding identity affirmation (Model 8).

Dependent Variable	Independent Variable	Coeff.	SE	t	LLCI	ULCI
Identity affirmation	AI relational roles	−1.19	0.26	−4.55 ***	−1.71	−0.68
	Goal distance	−1.06	0.26	−4.00 ***	−1.57	−0.54
	Int_1	0.78	0.17	4.74 ***	0.46	1.11
Participation intention	AI relational roles	−0.92	0.18	−5.13 ***	−1.27	−0.57
	Goal distance	−0.83	0.18	−4.65 ***	−1.18	−0.48
	Int_1	0.54	0.11	4.76 ***	0.32	0.76
	Identity affirmation	0.82	0.04	20.77 ***	0.74	0.89

Note: *** *p* < 0.001; Int_1 refers to the interaction between AI relational roles and goal distance.

**Table 5:** Mediating role of identity affirmation under different levels of goal distance.

Goal Distance	Effect	SE	LLCI	ULCI
Large-distance goal	−0.334	0.115	−0.573	−0.118
Small-distance goal	0.307	0.090	0.138	0.494

**Table 6:** Results of the mediating analysis regarding positive emotional resonance (Model 8).

Dependent Variable	Independent Variable	Coeff.	SE	t	LLCI	ULCI
Positive emotional resonance	AI relational roles	−1.48	0.31	−4.83 ***	−2.09	−0.88
	Goal distance	−1.37	0.31	−4.43 ***	−1.98	−0.76
	Int_1	0.98	0.19	5.07 ***	0.60	1.37
Participation intention	AI relational roles	−0.85	0.18	−4.79 ***	−1.20	−0.50
	Goal distance	−0.73	0.18	−4.11 ***	−1.08	−0.38
	Int_1	0.49	0.11	4.35 ***	0.27	0.71
	Positive emotional resonance	0.70	0.03	21.14 ***	0.64	0.77

Note: *** *p* < 0.001; Int_1 refers to the interaction between AI relational roles and goal distance.

**Table 7:** Mediating role of positive emotional resonance under different levels of goal distance.

Goal Distance	Effect	SE	LLCI	ULCI
Large-distance goal	−0.351	0.116	−0.589	−0.141
Small-distance goal	0.339	0.098	0.161	0.545

**Table 8:** Serial mediating role under different levels of goal distance.

Goal Distance	Effect	SE	LLCI	ULCI
Large-distance goal	−0.147	0.051	−0.255	−0.055
Small-distance goal	0.135	0.048	0.054	0.239

## Data Availability

The raw data supporting the conclusions of this article will be made available by the authors on request due to privacy concerns.
